# SYTL5 drives malignant progression in differentiated thyroid carcinoma: unveiling its regulatory mechanisms

**DOI:** 10.3389/fonc.2026.1810543

**Published:** 2026-09-03

**Authors:** Xiaohui Zhu, Xia Pan, Dongmei Jiang, Hengfeng Cui, Haiyan Wu, Yuying Wang, Xia Li

**Affiliations:** 1 Department of Endocrinology, Yancheng Third People’s Hospital, Yancheng, China; 2 Affiliated Hospital 6 of Nantong University, Yancheng, China; 3 Department of Breast and Thyroid Surgery, Yancheng Third People’s Hospital, Yancheng, China; 4 Department of General Practice, Yancheng Third People’s Hospital, Yancheng, China

**Keywords:** cell proliferation, oncogene, SYTL5 protein, thyroid carcinoma, ZNF384 protein

## Abstract

**Objective:**

To investigate the expression of SYTL5 in differentiated thyroid carcinoma (DTC) and elucidate its clinical significance and underlying molecular mechanisms.

**Methods:**

SYTL5 expression in 107 paired DTC and adjacent normal tissues was evaluated by immunohistochemistry, qRT-PCR, and Western blot. Associations with clinicopathological variables were assessed using chi-square or Fisher’s exact tests with Benjamini-Hochberg false discovery rate adjustment, and the distant metastasis subgroup was summarized descriptively because only one M1 case was available. Stable SYTL5 knockdown or overexpression DTC cell lines were established using RNA interference and lentiviral transfection. Cell proliferation, apoptosis, invasion, and migration were assessed using EdU staining, flow cytometry, and Transwell assays. A nude mouse subcutaneous xenograft model was constructed to evaluate tumor growth *in vivo*. Promoter dual-luciferase reporter assays were performed to evaluate transcriptional regulation of SYTL5 by ZNF384.

**Results:**

SYTL5 expression was significantly upregulated in DTC tissues and was positively associated with lymph node metastasis after false discovery rate adjustment. SYTL5 silencing inhibited DTC cell proliferation, invasion, and migration while modestly increasing apoptosis, whereas SYTL5 overexpression exerted opposite effects. Mechanistically, SYTL5 knockdown was associated with reduced TGFBR2 and phosphorylated Smad3 expression without affecting total Smad3 levels, both *in vitro* and *in vivo*. Promoter reporter assays demonstrated that ZNF384 suppressed wild-type SYTL5 promoter activity but not mutant promoter activity. ZNF384 overexpression suppressed SYTL5 expression, inhibited malignant phenotypes of DTC cells, and downregulated TGFBR2 and p-Smad3; these effects were partially reversed by SYTL5 overexpression.

**Conclusion:**

SYTL5 is associated with DTC progression and lymph node metastasis and functionally supports malignant phenotypes in DTC models. The data support a ZNF384-SYTL5-TGFBR2/Smad3 regulatory axis, while direct regulation of TGFBR2 by SYTL5 and the contribution of Smad2 or non-canonical pathways require further study. SYTL5 may represent a candidate therapeutic target for DTC.

## 1 Introduction

In recent years, thyroid carcinoma (TC) has emerged as the most common malignant tumor of the endocrine system, with an increasing incidence primarily attributed to the improved detection of papillary thyroid microcarcinomas (1). Differentiated thyroid carcinoma (DTC) constitutes the most common pathological subtype of TC. Although DTC generally has a favorable prognosis, certain subtypes (e.g., radioiodine-refractory DTC, RAIR-DTC) remain associated with high mortality rates (2). Therefore, identifying and investigating key genes implicated in DTC progression is critical for elucidating its underlying pathogenic mechanisms, controlling tumor progression, and developing novel therapeutic strategies.

SYTL5 exhibits diverse mechanisms of action and correlations across various tumor types. It is downregulated in adrenocortical carcinoma and positively correlated with patient overall survival (3). Furthermore, SYTL5 is upregulated in estrogen receptor-positive breast cancer cell lines and is involved in estrogen-mediated vesicular transport (4). Previous studies have demonstrated that SYTL5 promotes proliferation, invasion, and migration of papillary thyroid carcinoma cells; however, its function in other thyroid cancer cell lines, its effect on apoptosis, and the specific underlying mechanisms remain incompletely defined (5). We previously reported that SYTL5 is a potential biomarker and correlates with immune infiltrates in thyroid carcinoma (6). The present study explicitly builds on that report by focusing on an independent clinical cohort, apoptosis assays, TGFBR2/Smad3 signaling, promoter-level regulation by ZNF384, and *in vivo* rescue experiments, thereby separating the mechanistic validation performed here from the prior bioinformatics-oriented observations.

To further investigate the role of SYTL5 in TC, the present study was performed at the clinical, molecular, and *in vivo* levels. We analyzed SYTL5 expression and its correlation with clinicopathological characteristics in clinical samples, explored the underlying mechanisms using molecular techniques, and verified its functions through animal experiments, with the aim of clarifying SYTL5-mediated malignant biological behaviors and associated mechanisms in DTC.

## 2 Materials and methods

### 2.1 Patient samples

A total of 107 differentiated thyroid carcinoma (DTC) tissue samples were collected from inpatients who underwent surgery in the Department of Thyroid and Breast Surgery, Yancheng Third People’s Hospital, between January 2023 and December 2024. All samples were pathologically confirmed as DTC by two experienced pathologists according to standard histopathological criteria. No patients had received radiotherapy, chemotherapy, targeted therapy, or other adjuvant interventions prior to surgery, ensuring that the samples were not affected by exogenous therapeutic factors. Freshly resected tissue samples were immediately frozen in liquid nitrogen after excision and subsequently stored at -80°C (storage time <= 5 years). Patients were stratified into the SYTL5 low-expression group and SYTL5 high-expression group according to immunohistochemical scoring. Because this cohort was assembled from 2023 to 2024 and mature follow-up was not available, overall survival and disease-free survival were not prespecified endpoints. This study was approved by the Biomedical Ethics Committee of Yancheng Third People’s Hospital (approval number: LunShen-2022-04) and adhered to the ethical guidelines of the Declaration of Helsinki.

### 2.2 Cell lines

Human thyroid cancer cell lines (TPC-1, KTC-1, FTC-33, CAL-62), human normal thyroid cell line (Nthy-ori3-1), and 293T cells were purchased from Wuhan Pricella Biotechnology Co., Ltd. The BCPAP cell line was obtained from the Cell Bank of the Chinese Academy of Sciences. CAL-62 and 293T cells were cultured in high-glucose Dulbecco’s Modified Eagle Medium (DMEM; Gibco, USA), while the other cell lines were maintained in RPMI-1640 medium (Gibco, USA). All culture media were supplemented with 10% fetal bovine serum (FBS; Gibco, USA), 100 U/mL penicillin, and 100 μg/mL streptomycin. FTC-33 and CAL-62 were included for baseline expression screening, whereas TPC-1 and KTC-1 were selected for functional experiments because they showed reproducible SYTL5 modulation and stable growth/transfection performance under the experimental conditions.

### 2.3 Animal sources

A total of 25 SPF-grade female BALB/c nude mice (athymic immunodeficient strain), aged 4–6 weeks and weighing 18–20 g (weight difference within ±2 g), were provided by Shanghai SLAC Laboratory Animal Co., Ltd. (production license number: SCXK (Hu) 2017-0005). The mice were housed in the animal facility of Jiangsu Vocational College of Medicine, an SPF-grade barrier environment in compliance with the national standard *Laboratory Animal Environment and Facilities* (GB 14925-2010). The environment was controlled by a central air-conditioning system, maintained at a temperature of 21–25°C (daily fluctuation ≤ 2°C) and a relative humidity of 65%–70% (daily fluctuation ≤ 5%) with dehumidification and humidification equipment. A 12-hour light/dark cycle was strictly followed (lighting period: 8:00–20:00). The experimental protocol was approved by the Institutional Animal Ethics Committee (approval number: XMLL-2022-863) and strictly adhered to the 3R principles (Replacement, Reduction, Refinement).

### 2.4 Transient transfection of shRNA

Cells in the logarithmic growth phase were trypsinized, resuspended in complete medium, and counted using a hemocytometer. Cells were seeded into 6-well plates at a density of 5×10^5^–8×10^5^ cells per well, supplemented with 2 mL of complete medium, and cultured at 37°C with 5% CO_2_ until confluency reached 50%–70%. shRNA sequences targeting SYTL5 were synthesized as follows: SYTL5-sh#1: ATAGACCTGGGCGTCCTAAAG, SYTL5-sh#2: ATTGTGTGTGTCCAGAGATCATT, SYTL5-sh#3: GGAACTGACGGTCAACATTT, and negative control: TTCTCGAACGTCACGT. The original medium in the 6-well plate was discarded, and cells were washed once with 1 mL of phosphate-buffered saline (PBS), followed by the addition of 1 mL of serum-free medium per well. Pre-incubated shRNA-transfection reagent complexes were added dropwise to the wells, and the plate was gently shaken to ensure uniform distribution. Cells were cultured at 37°C with 5% CO_2_ for 4–6 hours. Following transfection, the serum-free medium was replaced with 2 mL of complete medium supplemented with 10% FBS, and cell culture was continued.

### 2.5 Construction of stable cell lines

Lentiviral packaging and stable cell line construction were performed in 293T cells. One day prior to transfection, 293T cells were seeded into 6-well plates at 5×10^5^ cells per well. When cell confluency reached 70%–80%, 3 μg of target vector, 2 μg of psPAX2, and 1 μg of pMD2.G were mixed in 150 μL of Opti-MEM to prepare Solution A. Meanwhile, 10 μL of transfection reagent was diluted in the same volume of Opti-MEM to prepare Solution B. After standing at room temperature for 5min, the two solutions were mixed and incubated for an additional 20min, then added dropwise to the wells. Cells were cultured at 37°C with 5% CO_2_. The medium was discarded after 24h and replaced with 3 mL of complete medium. Supernatants were first collected after 24h of culture and again at 48h. The supernatants were centrifuged at 3000 rpm for 10min at 4°C and filtered through a 0.45 μm membrane to remove debris. If necessary, the virus was concentrated by ultracentrifugation at 25,000 rpm for 2h at 4°C or PEG-8000 precipitation, and finally resuspended in 100–200 μL of PBS and stored at -80°C in aliquots. Viral titer was determined by gradient dilution: the virus solution was serially diluted from 10^-^¹ to 10^-6^ and used to infect 1×10^4^ 293T cells in 24-well plates with the addition of 8 μg/mL Polybrene. After 48h, 2 μg/mL puromycin was added for selection for 7–10 days. Resistant clones were counted, and the titer was calculated as (number of clones × dilution factor × well volume)/number of seeded cells to determine the multiplicity of infection (MOI). The minimum effective MOI was determined by pre-experiment: 1×10^4^ cells per well were seeded into 24-well plates, and virus solutions with MOI ranging from 0.1 to 100 were added with Polybrene. The medium was replaced after 24h, and antibiotics were added after 48h until all control cells died. For formal infection, 5×10^5^ cells per well were seeded into 6-well plates, and virus solution with the optimal MOI and Polybrene were added. The medium was replaced with complete medium after 24h, and antibiotic-containing medium was changed every 2–3 days for 2 weeks until all control cells died. Surviving clones were trypsinized and serially diluted into 96-well plates for single-cell culture. The expression of target genes and proteins in expanded monoclonals was verified by quantitative real-time polymerase chain reaction (qRT-PCR) and Western blot (WB).

### 2.6 Quantitative real-time PCR

Tissue samples from patients were rinsed with pre-cooled PBS, minced, and ground into powder in liquid nitrogen. 1 mL of Trizol reagent was added to 50–100 mg of tissue, followed by vortexing. For adherent cells, the medium was discarded, cells were gently washed twice with calcium- and magnesium-free PBS, and 1 mL of Trizol reagent was added per 10 cm² of culture dish. After standing at room temperature for 5min, cells were pipetted until the solution became clear and transferred to RNase-free EP tubes. Chloroform (0.2 mL) was added at a Trizol:chloroform ratio of 5:1, and the mixture was vigorously shaken for 15 seconds, followed by standing at room temperature for 2–3min. After centrifugation at 12,000 rpm for 15min at 4°C, 0.4–0.6 mL of the upper aqueous phase was carefully collected, mixed with an equal volume of isopropanol, and incubated at room temperature for 10min. RNA pellets were obtained by centrifugation at 12,000 rpm for 10min. The supernatant was discarded, and the pellets were resuspended in 1 mL of pre-cooled 75% ethanol (prepared with DEPC water) by vortexing, followed by centrifugation at 7500 rpm for 5min at 4°C. The washing step was repeated, and the pellets were air-dried at room temperature, then dissolved in 30–50 μL of DEPC water by incubating at 55–60°C for 5min. RNA samples were stored at -80°C or immediately detected using a NanoDrop spectrophotometer. Samples with an OD260/280 ratio between 1.8 and 2.0 and concentration = OD260 × dilution factor × 40 μg/mL were used for reverse transcription. The PCR instrument was preheated at 65°C, and the PCR reagents were mixed and incubated in the PCR instrument as follows: 25°C for 10 min → 42°C for 60 min → 70°C for 10 min → 4°C. The resulting cDNA was either used directly for PCR or stored at -80°C for long-term use. Subsequent qRT-PCR was performed using the synthesized cDNA. The primer sequences for each target gene are as follows: - SYTL5: F: ATGTTCTCGGCACTGATGTTGTCC, R: CGGGTTTGCTCTTGGCTCTGTAC; ZNF384: F: CACCACCACACTTCCAGTCTCCTG, R: GGTGACAGTGAGGCAGATGTCCTT; β-actin: F: CATGTACGTTGCTATCCAGGC, R: CTCCTTAATGTCACGCACGAT.

### 2.7 Western blot

Twenty to fifty milligrams of thyroid cancer and adjacent tissues from patients or confluent cells in 60 mm dishes were lysed. Protein concentration was determined using the bicinchoninic acid (BCA) method. Twenty micrograms of protein was mixed with loading buffer and denatured at 100°C for 5 min. After electrophoresis and transfer to a polyvinylidene difluoride membrane, membranes were blocked with 5% non-fat milk powder at room temperature for 2 h and incubated with primary antibodies overnight at 4°C. The primary antibody information was as follows: SYTL5 (Abcam, #203218, dilution: 1:1000), TGFBR2 (Abcam, #ab61213, dilution: 1:1000), ZNF384 (Abcam, #ab176689, dilution: 1:1000), Smad3 (CST, #9523, dilution: 1:1000), p-Smad3 (CST, #12446, dilution: 1:1000), and β-actin (CST, #4970, dilution: 1:1000). Horseradish peroxidase-conjugated secondary antibodies were added and incubated at room temperature for 1 h. Enhanced chemiluminescence reagent was used for visualization, and ImageJ software was employed to calculate the gray value ratio of target proteins to internal reference for relative quantification.

### 2.8 Immunohistochemistry

One hundred and seven DTC samples and paired adjacent tissues fixed with 4% paraformaldehyde for 24h were sent to Wuhan Servicebio Technology Co., Ltd. for paraffin embedding, orientation, and tissue microarray construction: 2 points (φ1.5 mm × 4 μm) were taken from each lesion and adjacent tissue, totaling 428 dots. Paraffin blocks were pre-cooled at -20°C for 30min, then sectioned continuously using a Leica RM2235 microtome. Sections were spread in 45°C warm water, mounted on poly-L-lysine-coated glass slides, baked at 60°C for 2h, and stored at 4°C. Subsequent procedures were performed as follows: deparaffinization and hydration through xylene I–III for 10min each, 100% ethanol for 5min twice, and 95% → 85% → 75% ethanol for 3min each; rinsed with PBS 3 times for 5min each; microwave antigen retrieval in 0.01 M citrate buffer (pH 6.0) for 30min, followed by natural cooling and PBS rinsing; blocking of endogenous peroxidase with 3% H_2_O_2_-methanol at room temperature in the dark for 15min, then PBS rinsing 3 times for 5min each; circling the tissue with an immunohistochemical pen and blocking with 5% bovine serum albumin (BSA) at 37°C for 30min. The blocking solution was discarded, and 1:200 diluted rabbit anti-human SYTL5 polyclonal antibody was added directly, followed by incubation overnight at 4°C in a humid chamber. The next day, the slides were rewarmed at room temperature for 30min and rinsed with PBS 3 times for 5min each. HRP-conjugated goat anti-rabbit IgG polymer was added and incubated at 37°C for 30min, then rinsed with PBS 3 times for 5min each. DAB chromogen (A:B = 1:20) was added, and color development was controlled under a microscope for 30–90 seconds, followed by termination with running water. Hematoxylin counterstaining was performed for 30 seconds, differentiation with 1% hydrochloric acid alcohol for 3 seconds, and bluing with 1% ammonia water for 10 seconds, then rinsed with running water for 5min. Dehydration was carried out through gradient ethanol (75% → 85% → 95% → 100%) for 2min each, clearing with xylene twice for 5min each, and mounting with neutral balsam. Slides were independently reviewed by two associate chief physicians in a double-blind manner. SYTL5 was localized in the cytoplasm/membrane, and brownish-yellow granules were considered positive. The scoring criteria were as follows: positive cells ≤5%: 0 points, 6–25%: 1 point, 26–50%: 2 points, 51–75%: 3 points, >75%: 4 points; staining intensity: no staining: 0 points, light yellow: 1 point, brownish-yellow: 2 points, dark brown: 3 points. A product of the two scores ≥4 was defined as high expression, and <4 as low expression. Discrepancies were resolved through consultation (7).

### 2.9 Transwell assay

Cells in the logarithmic growth phase were trypsinized at 37°C for 1–2min, terminated with complete medium, centrifuged at 1000 rpm for 5min, washed 1–2 times with PBS, resuspended in serum-free RPMI-1640 medium, and counted. For the migration assay, 200 μL of cell suspension (density adjusted according to pre-experiment) was directly added to the upper chamber of an 8 μm pore Transwell insert. For the invasion assay, Matrigel was diluted 1:4 with serum-free medium on ice, 40 μL was evenly spread on the bottom of the upper chamber, gelled at 37°C for 2h, and hydrated with 50 μL of serum-free medium for 30min before seeding cells at the same volume. The lower chamber of the 24-well plate was filled with 600 μL of complete medium containing 15% FBS as a chemoattractant. The insert was gently placed into the well to eliminate air bubbles and incubated at 37°C with 5% CO_2_ for 24h (48h for the invasion assay). After incubation, the insert was removed, washed twice with PBS, and cells on the upper surface of the membrane were gently wiped off with a moist cotton swab. Cells were fixed with 4% paraformaldehyde for 30min (15min for the invasion assay), air-dried at room temperature, and stained with 0.1% crystal violet for 30–60min. Excess dye was rinsed off with running water, and cells on the lower surface of the membrane were counted under an inverted microscope at 400× magnification in 5 randomly selected fields. The average number of cells was used to evaluate cell migration and invasion abilities.

### 2.10 Flow cytometry for apoptosis detection (Annexin V/PI double staining)

Cell suspensions (5×10^5^ cells/500 μL) were collected into flow cytometry tubes. 5 μL of Annexin V-FITC and 10 μL of propidium iodide (PI) were added sequentially, gently vortexed, and incubated at room temperature in the dark for 15min. Immediately afterward, 400 μL of 1× Binding Buffer was added to a final volume of 900 μL, gently pipetted, and analyzed by flow cytometry. The instrument was excited with a 488 nm laser, with FL1 (530/30 nm) detecting green fluorescence and FL2/FL3 (615/20 nm) detecting red fluorescence. At least 1×10^4^ viable cells were acquired at a low speed of 500 events/s. Compensation was adjusted using single-stained tubes, and quadrants were set as follows: Annexin V^-^/PI^-^ (Q3: viable cells), Annexin V^+^/PI^-^ (Q4: early apoptosis), Annexin V^+^/PI^+^ (Q2: late apoptosis/necrosis), and Annexin V^-^/PI^+^ (Q1: necrosis). The total apoptosis rate was calculated as (Q4 + Q2)/total cells × 100%.

### 2.11 EdU cell proliferation assay

The culture medium was discarded, and cells were gently washed twice with PBS. 1 mL of fixative was added to each well and incubated at room temperature for 15min. After discarding the fixative, cells were washed 3 times with PBS for 3min each. Subsequently, 1 mL of permeabilization solution was added and incubated at room temperature for 15min, followed by rinsing with PBS twice for 5min each. Click Additive was dissolved in 1.3 mL of deionized water to prepare the reaction solution, and 0.5 mL was added to each well. The reaction was carried out in a humid chamber at room temperature in the dark for 30min, and cells were washed 3 times with PBS for 5min each with shaking. Endogenous peroxidase was blocked with 1 mL of blocking solution at room temperature for 20min, and cells were gently washed 3 times with PBS for 2min each. Streptavidin-HRP was diluted 1:100 with PBS, 200 μL was added to each well, and incubated in a humid chamber at room temperature in the dark for 30min. After washing 3 times with PBS for 2min each, 200 μL of working solution (equal volumes of DAB A and B solutions mixed) was added to each well, and color development was performed at room temperature in the dark for 5–30min. The solution was discarded when brownish-yellow granules were observed under the microscope, and the reaction was terminated by washing 3 times with PBS. EdU-positive proliferating cells were photographed and recorded directly under a microscope.

### 2.12 Dual-luciferase reporter gene assay

To verify the transcriptional regulation of SYTL5 by ZNF384, dual-luciferase reporter plasmids containing the wild-type (WT) and mutant (MUT) SYTL5 promoter fragments encompassing the predicted ZNF384-binding motif were synthesized. TPC-1 cells in the logarithmic growth phase were seeded into 24-well plates at 5×10^4^ cells per well. When confluency reached 70%-80%, the medium was replaced with serum-free DMEM for equilibration. Subsequently, 500 ng of WT or MUT promoter reporter plasmid was mixed with 500 ng of ZNF384 overexpression or empty vector plasmid in 100 μL of serum-free DMEM to prepare the plasmid mixture. Meanwhile, 2 μL of Lipofectamine 2000 was diluted in the same volume of serum-free DMEM and incubated at room temperature for 20 min to form complexes, which were then added dropwise to the wells. The medium was replaced with complete medium containing 10% FBS after 6 h. At 48 h post-transfection, cells were washed twice with pre-cooled PBS, and 100 μL of 1× Cell Lysis Buffer was added to each well. Cells were lysed on a shaker at room temperature for 5 min, and the lysate was collected by pipetting and centrifuged at 11,200 rpm for 2 min to collect the supernatant. Twenty microliters of supernatant was mixed with 100 μL of firefly luciferase substrate to measure RLU1, followed by the addition of 100 μL of Renilla luciferase substrate to measure RLU2. The relative luciferase activity was calculated as the ratio of RLU1/RLU2. Each group had 3 replicate wells, and the experiment was repeated 3 times. The promoter regulatory effect of ZNF384 on SYTL5 was determined by comparing WT and MUT promoter reporter activity (8).

### 2.13 Nude mouse subcutaneous xenograft assay

TPC-1 cells in the logarithmic growth phase were trypsinized, resuspended in serum-free medium, and cell viability was confirmed to be >90% by trypan blue staining. The cell concentration was adjusted to 1×10^7^ cells/mL. SPF-grade BALB/c nude mice were anesthetized intraperitoneally with pentobarbital sodium (50 mg/kg), and the axillary regions were disinfected with 75% ethanol. 100 μL (1×10^6^ cells) of cell suspension was subcutaneously injected into each axilla using a 1 mL syringe, and the injection site was gently pressed to prevent leakage. The general status of the mice was observed daily, and the bedding was changed twice a week. Sterilized feed and drinking water were provided ad libitum. Subcutaneous nodules were palpable in 100% of the mice within 7–14 days. Starting from the appearance of nodules, the long diameter (L) and short diameter (W) of the tumors were measured every 3 days with a vernier caliper, and the tumor volume was calculated using the formula V = ½ × L × W². Growth curves were plotted accordingly. On day 21, the mice were euthanized by cervical dislocation, and the tumors were immediately dissected completely, weighed, and measured. The tumor tissues were minced, aliquoted, frozen in liquid nitrogen, and transferred to -80°C for subsequent RNA and protein analysis (9).

### 2.14 Statistical analysis

Western blot and immunohistochemical images were processed using Adobe Photoshop 2020. The expression level of SYTL5 in IHC was determined based on staining intensity. Gray values of Western blot bands were analyzed using ImageJ software. All statistical analyses were performed using GraphPad Prism 8.0 software. Experimental data were derived from at least 3 independent repeated experiments and expressed as mean ± standard deviation (Mean ± SD). The association between SYTL5 expression levels and clinicopathological parameters was analyzed using Pearson’s chi-square test or Fisher’s exact test when expected counts were <5. Benjamini-Hochberg false discovery rate (FDR) adjustment was applied to the clinicopathological comparisons of age, gender, T stage, N stage, and TNM stage. M stage was summarized descriptively and excluded from formal comparative testing because the distant metastasis subgroup contained only one patient. Normality of data was tested using the Shapiro-Wilk test (n <= 50) or Kolmogorov-Smirnov test (n > 50). Homogeneity of variance was assessed by Levene’s test. For comparison between two groups, the independent samples t-test was used for normally distributed data with homogeneous variance, and the Mann-Whitney U test was used for non-normally distributed data or heterogeneous variance. For comparison among multiple groups, one-way analysis of variance followed by Tukey’s *post-hoc* test was used. A P-value <0.05 was considered statistically significant; for clinicopathological comparisons, both raw and FDR-adjusted P values were reported.

## 3 Results

### 3.1 SYTL5 is highly expressed in thyroid carcinoma and associated with lymph node metastasis

To further characterize SYTL5 expression in DTC, immunohistochemistry (IHC) was used to assess SYTL5 protein expression in thyroid cancer tissues and adjacent normal tissues from 107 DTC patients admitted to Yancheng Third People’s Hospital between January 2023 and December 2024. The clinicopathological characteristics of the thyroid cancer patients are summarized in **Supplementary Table 1**. Among the patients, there were 22 males and 85 females, including 105 cases of papillary carcinoma and 2 cases of follicular carcinoma. Of all 107 DTC patients, 74 were aged 45 years or older. There was no significant difference in the high expression rate of SYTL5 between patients aged <45 years (60.6%, 20/33) and those aged >=45 years (56.8%, 42/74) (raw P = 0.710, FDR-adjusted P = 0.717). Similarly, no significant difference was observed between males (54.5%, 12/22) and females (58.8%, 50/85) (raw P = 0.717, FDR-adjusted P = 0.717). The high expression rate of SYTL5 in patients with T1–2 stage tumors (60.9%, 56/92) was not significantly different from that in patients with T3–4 stage tumors (40.0%, 6/15) (raw P = 0.129, FDR-adjusted P = 0.323). However, the high expression rate of SYTL5 in patients with lymph node metastasis (75.6%, 31/41) was significantly higher than that in patients without lymph node metastasis (47.0%, 31/66) (raw P = 0.0035, FDR-adjusted P = 0.0175). Distant metastasis was observed in one patient (1/107) and was therefore summarized descriptively without formal comparative testing. No significant difference was found between patients with TNM stage I-II (58.7%, 61/104) and stage III-IV (33.3%, 1/3) (raw P = 0.381, FDR-adjusted P = 0.635).

**Figure 1:**
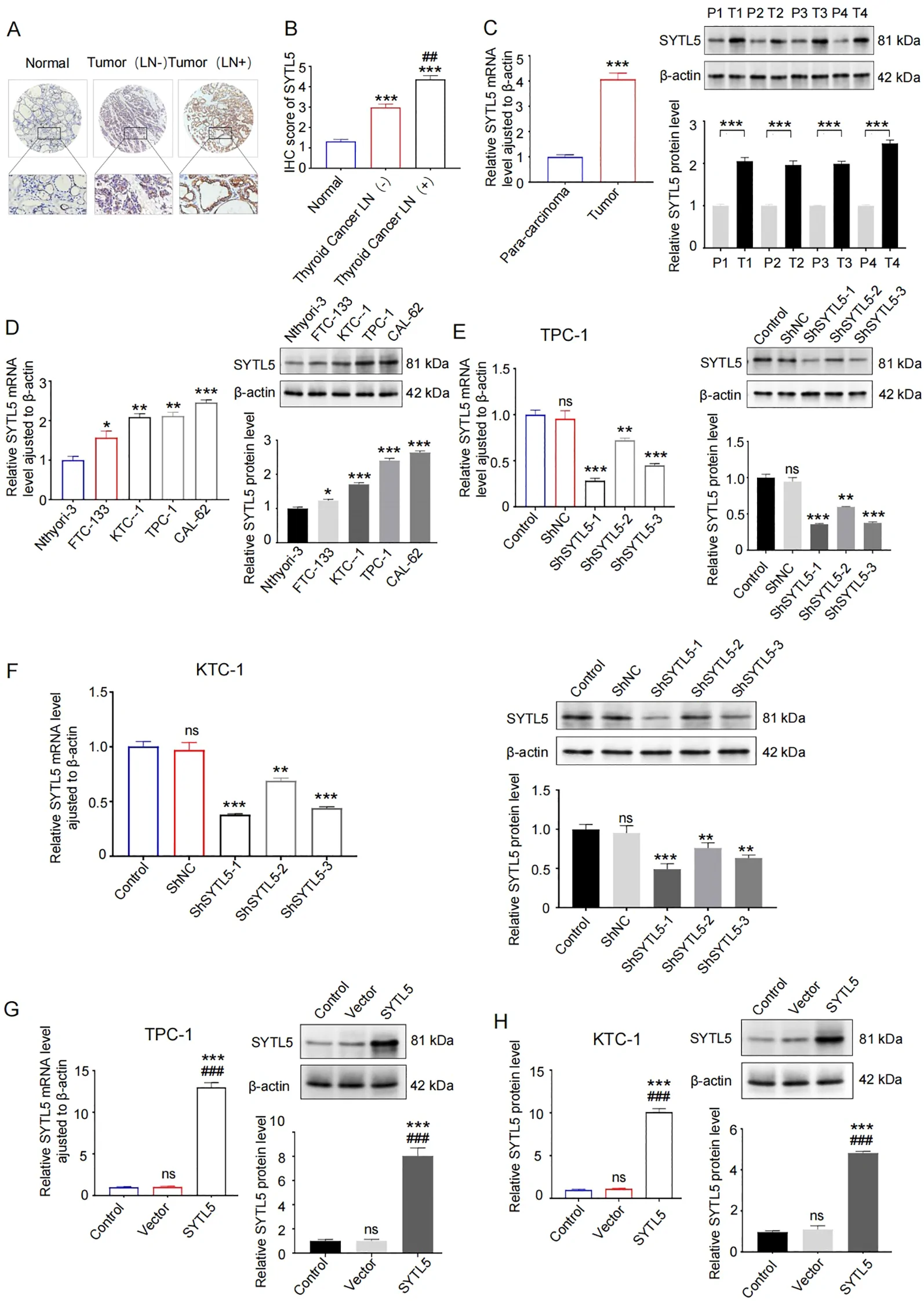
Correlation between SYTL5 expression and clinicopathological characteristics. **(A)** IHC results showing the difference in SYTL5 expression among adjacent normal tissues, primary DTC tissues without lymph node metastasis, and primary DTC tissues with lymph node metastasis (200×). **(B)** Correlation between IHC scores of SYTL5 expression and lymph node metastasis in differentiated thyroid carcinoma. **(C)** qPCR and Western blot verification of SYTL5 mRNA and protein expression levels in differentiated thyroid carcinoma tissues and adjacent normal tissues. **(D)** qPCR and Western blot verification of SYTL5 mRNA and protein expression levels in thyroid follicular epithelial cells and thyroid cancer cell lines used for baseline screening. **(E, F)** qRT-PCR and Western blot verification of successful silencing of SYTL5 mRNA and protein in TPC-1 and KTC-1 cells. **(G, H)** qRT-PCR and Western blot verification of successful overexpression of SYTL5 in TPC-1 and KTC-1 cells. (*P<0.05, **P<0.01, ***P<0.001, compared with the control group; ##P<0.01, ###P<0.001, compared with the vector group; ns, no statistical difference).

Comparison of SYTL5 expression among adjacent normal tissues, primary tumor tissues without lymph node metastasis, and primary tumor tissues with lymph node metastasis by IHC revealed that SYTL5 protein expression was significantly higher in DTC patients with lymph node metastasis than in those without (**Figure 1A**). Moreover, IHC scoring results showed that the SYTL5 score was significantly higher in patients with lymph node metastasis than in the non-metastatic group (P<0.001, **Figure 1B**). These findings suggest that high SYTL5 expression in primary DTC tissues is associated with the lymph node metastatic phenotype. Metastatic lymph node deposits were not included in the tissue microarray; therefore, SYTL5 expression in matched lymph node metastatic sites could not be quantified directly in this cohort.

We further verified the expression of SYTL5 in tissues and cells. Four pairs of DTC tissues were randomly selected for qRT-PCR and Western blot to verify the mRNA and protein expression of SYTL5. The results showed that SYTL5 expression was significantly upregulated in DTC tissues (**Figure 1C**). Consistent with these findings, the mRNA and protein levels of SYTL5 in thyroid cancer cell lines (KTC-1, FTC-33, TPC-1, CAL-62) were higher than those in the non-tumor thyroid follicular epithelial cell line Nthy-ori3-1 (**Figure 1D**). FTC-33 and CAL-62 were used for expression screening, whereas TPC-1 and KTC-1 were selected for subsequent functional experiments. For functional experiments, SYTL5-silenced and overexpressed cell lines were constructed, and transfection efficiency was confirmed by qRT-PCR and Western blot. The results showed that SYTL5 expression was significantly decreased in TPC-1 and KTC-1 cells after transfection with sh-SYTL5 (**Figures 1E, F**). sh-SYTL5–1 was selected as the target gene knockdown construct for further experiments. The overexpression efficiency of SYTL5 was also detected, and the results showed that after transfection with SYTL5, the protein expression level of SYTL5 was significantly higher than that in the control group (P<0.001), indicating successful construction of the overexpression model (**Figures 1G, H**).

### 3.2 SYTL5 affects the proliferation and apoptosis of DTC cell lines

**Figure 2:**
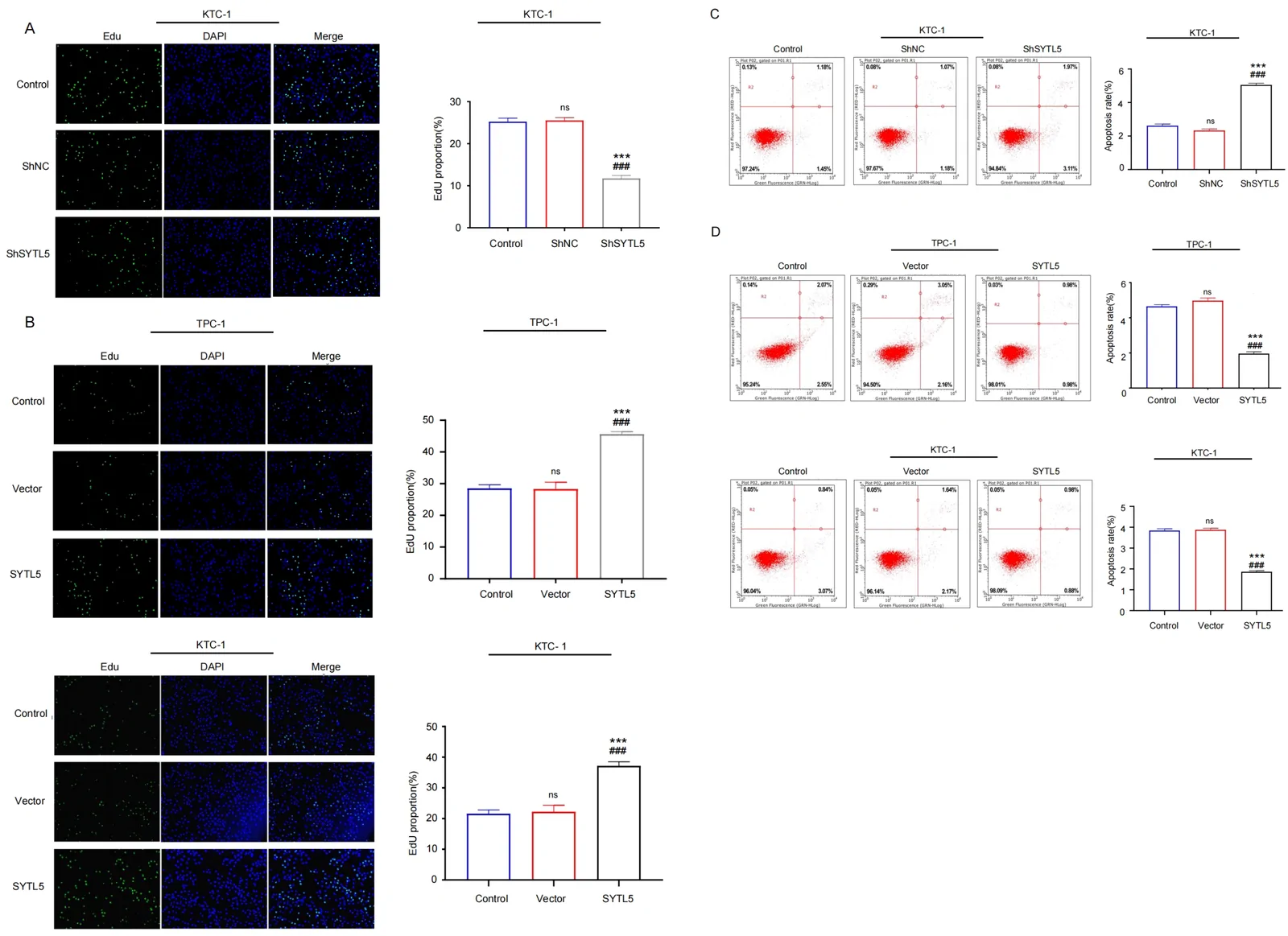
SYTL5 affects the proliferation and apoptosis of DTC cell lines. **(A, B)** EdU staining results showing the effect of SYTL5 silencing and overexpression on DTC cell proliferation (100×). **(C, D)** Annexin V-FITC/PI double staining flow cytometry results showing the effect of SYTL5 silencing or overexpression on DTC cell apoptosis. The apoptosis changes were statistically significant but modest in absolute magnitude and were interpreted together with proliferation and motility data. (***P<0.001, compared with the control group; ###P<0.001, compared with the vector group; ns, no statistical difference).

Normal cells are strictly regulated with a balance between proliferation and apoptosis; however, tumor cells disrupt this balance due to various factors, acquiring sustained proliferative capacity. Uncontrolled proliferation is a core driver of tumor progression from benign to malignant, as it not only determines tumor growth rate but also promotes invasion and metastasis by inducing angiogenesis and remodeling the microenvironment. To evaluate the effect of SYTL5 on the proliferation of differentiated thyroid carcinoma, EdU assays were performed to determine the proliferation of DTC cells after SYTL5 silencing or overexpression. The results showed that the proportion of EdU-positive KTC-1 cells was significantly lower in the SYTL5-silenced group than in the control group (P<0.001) (**Figure 2A**). In contrast, the proportion of EdU-positive TPC-1 and KTC-1 cells was significantly higher in the SYTL5-overexpressed group than in the control group (P<0.001) (**Figure 2B**). These results suggest that SYTL5 can promote the DNA synthesis and proliferative activity of DTC cells.

Abnormal cell apoptosis is an important characteristic of tumor development. Tumor cells evade apoptosis, enabling them to sustain proliferation and escape immune surveillance. To confirm whether SYTL5 is involved in tumor progression by regulating cell apoptosis, flow cytometry was used to analyze the effect of SYTL5 on cell apoptosis. Annexin V-FITC/PI double staining flow cytometry showed that the apoptosis rate of KTC-1 cells was significantly increased after SYTL5 silencing (P<0.001) (**Figure 2C**). The absolute increase in apoptosis was modest, rising from approximately 2.6% in control cells to approximately 5.0% after SYTL5 silencing, while more than 90% of cells remained viable. Therefore, SYTL5 silencing was interpreted as producing a reproducible but limited pro-apoptotic effect rather than apoptosis being the sole driver of the observed functional phenotype. In contrast, the apoptosis rate of TPC-1 and KTC-1 cells was decreased after SYTL5 overexpression (P<0.001) (**Figure 2D**). These results indicate that SYTL5 modestly suppresses apoptosis in DTC cells, in parallel with its stronger effects on proliferation, invasion, and migration.

### 3.3 SYTL5 affects the invasive and migratory capacities of DTC cell lines

**Figure 3:**
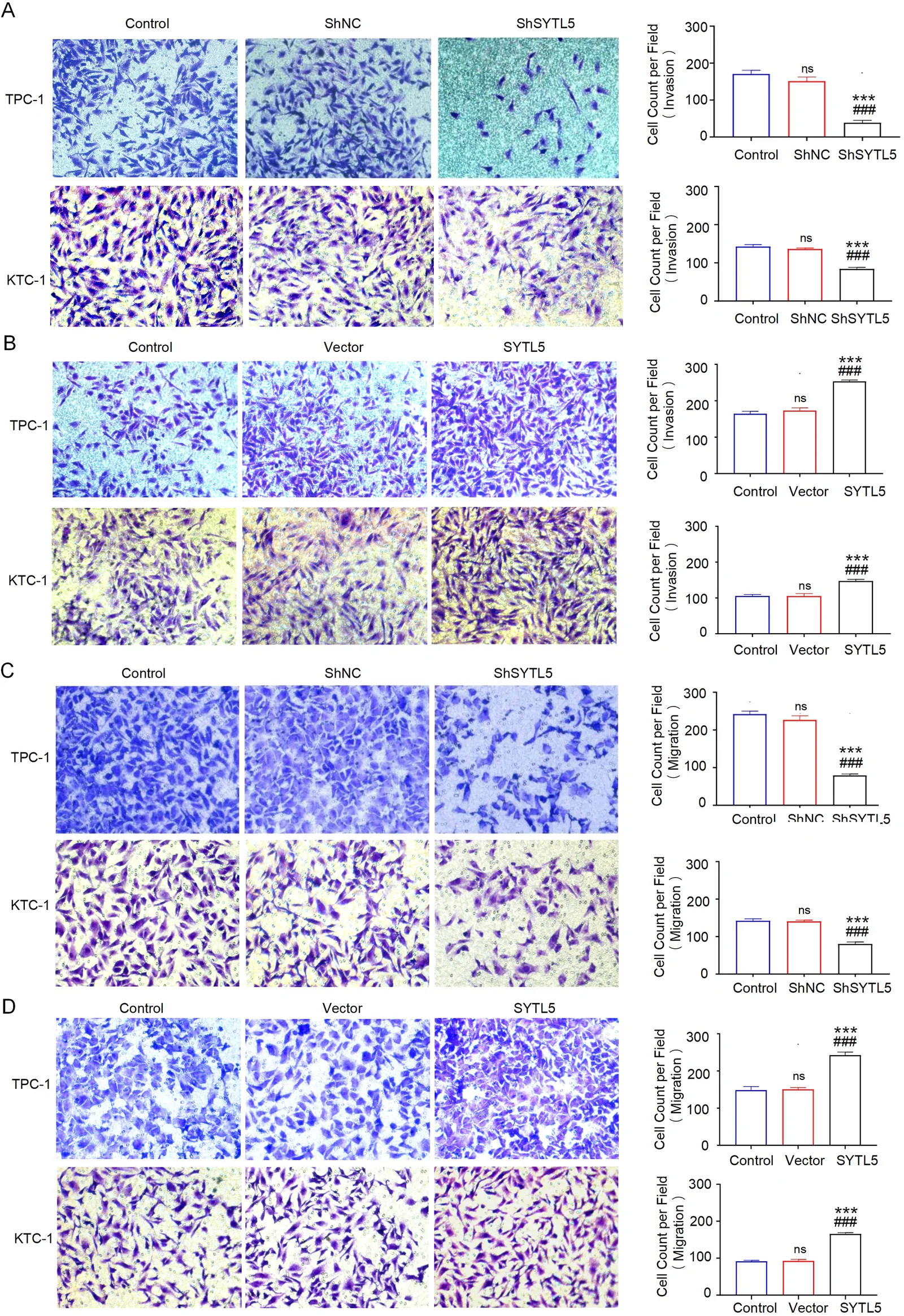
SYTL5 affects the invasive and migratory capacities of DTC cell lines. **(A, B)** Transwell assay results showing the effect of SYTL5 silencing and overexpression on the invasive capacity of DTC cells (200×). **(C, D)** Transwell assay results showing the effect of SYTL5 silencing and overexpression on the migratory capacity of DTC cells (200×). (***P<0.001, compared with the control group; ###P<0.001, compared with the vector group; ns, no statistical difference).

Invasion and migration are key steps in malignant tumor progression, exerting a decisive impact on tumor metastasis, prognosis, and therapeutic resistance. To verify the effect of SYTL5 on DTC invasion and migration, Transwell assays were performed. Invasion assay results showed that the number of transmembrane cells of TPC-1 and KTC-1 was significantly reduced after SYTL5 silencing compared with the control group (215 ± 23) (P<0.001) (**Figure 3A**), while the number of transmembrane cells was significantly increased after SYTL5 overexpression (P<0.001) (**Figure 3B**). Migration assay results showed that compared with the control group, the number of transmembrane cells of TPC-1 and KTC-1 was significantly reduced after SYTL5 silencing (P<0.001) (**Figure 3C**), and significantly increased after SYTL5 overexpression (P<0.001) (**Figure 3D**). These results indicate that SYTL5 promotes the invasion and migration of DTC cells.

### 3.4 SYTL5 knockdown is associated with reduced TGFBR2/Smad3 signaling in DTC cells

**Figure 4:**
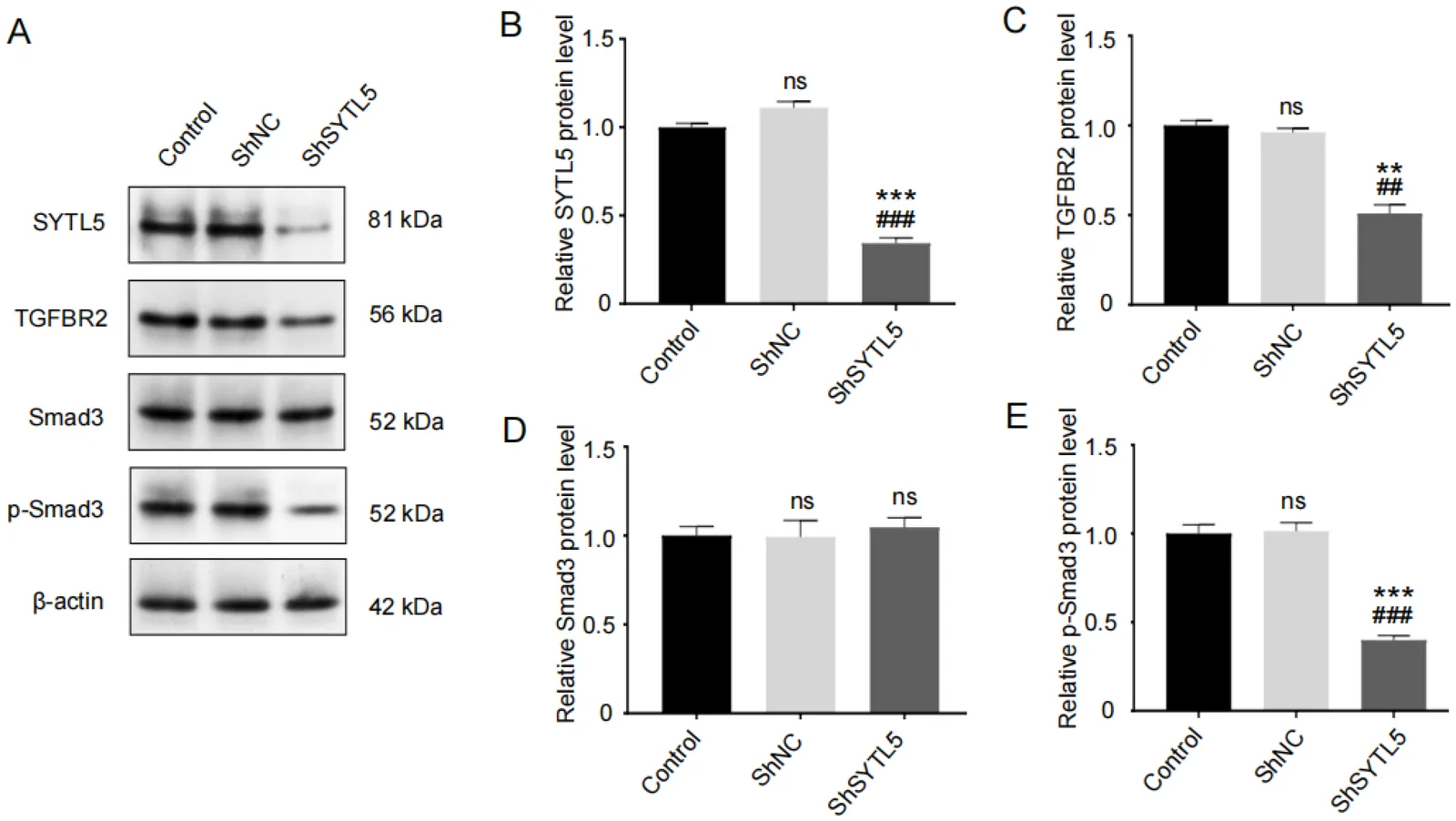
SYTL5 knockdown is associated with reduced TGFBR2/Smad3 signaling in DTC cells. **(A)** Protein expression levels of SYTL5, TGFBR2, Smad3, and p-Smad3 in SYTL5-silenced TPC-1 cells. **(B)** Quantitative analysis of SYTL5 protein in SYTL5-silenced TPC-1 cells. **(C)** Quantitative analysis of TGFBR2 protein in SYTL5-silenced TPC-1 cells. **(D)** Quantitative analysis of Smad3 protein in SYTL5-silenced TPC-1 cells. **(E)** Quantitative analysis of p-Smad3 protein in SYTL5-silenced TPC-1 cells. (**P<0.01, ***P<0.001, compared with the control group; ##P<0.01, ###P<0.001, compared with the shNC group; ns, no statistical difference).

The TGF-β signaling pathway plays a dual regulatory role in tumor development and progression, and abnormal activation of its downstream Smad signaling is closely associated with tumor cell proliferation and invasion. In previous experiments, SYTL5 was found to be involved in malignant biological behaviors of DTC cells, including proliferation, invasion, and migration. Previous studies have confirmed that the TGF-β signaling pathway is related to the tumor immune microenvironment (7, 8), and our preliminary bioinformatics analysis also revealed a correlation between SYTL5 and immune infiltration. Therefore, Western blot assays were performed to detect the expression of key molecules in the TGF-β pathway in SYTL5-silenced DTC cells. The results showed that compared with the control group, the protein levels of SYTL5, TGFBR2, and p-Smad3 were significantly decreased in SYTL5-silenced DTC cells (P<0.001), while total Smad3 levels remained unchanged (**Figures 4A–E**). These results suggest that SYTL5 is associated with TGFBR2 abundance and downstream Smad3 phosphorylation. Because no promoter-binding assay or chromatin immunoprecipitation experiment was performed for TGFBR2, the current data support pathway-level modulation rather than direct transcriptional regulation of TGFBR2 by SYTL5.

### 3.5 *In vivo* experiments support the association between SYTL5 and TGFBR2/Smad3 signaling during xenograft growth

**Figure 5:**
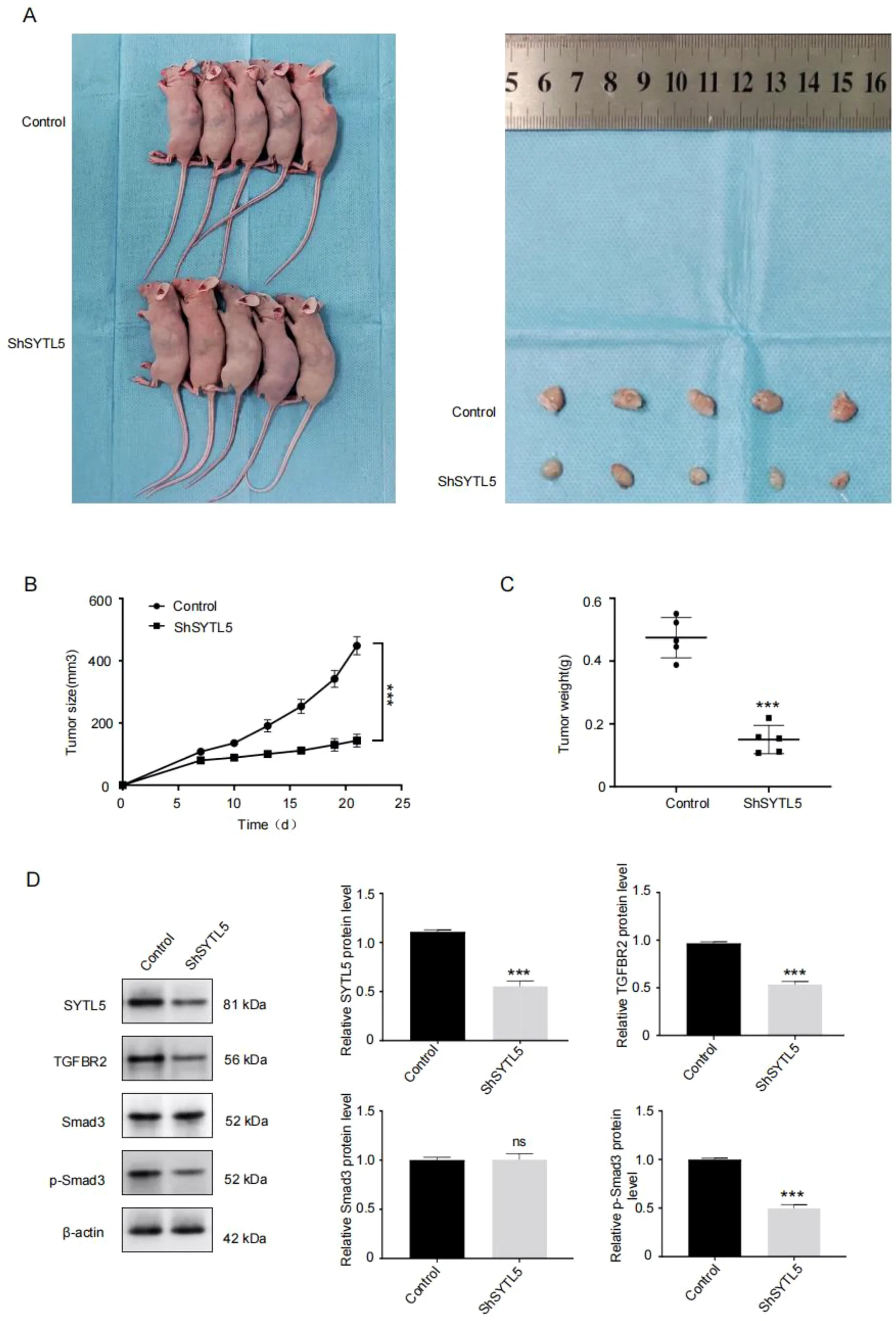
*In vivo* experiments support the association between SYTL5 and TGFBR2/Smad3 signaling during thyroid carcinoma xenograft growth. **(A)** Effect of SYTL5 silencing on the size of TPC-1 subcutaneous xenografts. **(B)** Effect of SYTL5 silencing on the volume of TPC-1 subcutaneous xenografts. **(C)** Effect of SYTL5 silencing on the weight of TPC-1 subcutaneous xenografts. **(D)** Effect of SYTL5 silencing on SYTL5 mRNA and TGFBR2/Smad3 pathway molecule protein expression levels in TPC-1 subcutaneous xenografts. (***P<0.001, compared with the control group; ns, no statistical difference).

To verify the effect of SYTL5 on DTC cells *in vivo*, control group and SYTL5-silenced TPC-1 cells were subcutaneously injected into the axillae of nude mice (5 mice per group). Tumor volume was measured every 3 days after inoculation. The results showed that the growth rate of subcutaneous xenografts in the SYTL5-silenced group was significantly slower than that in the control group, and the average tumor volume and weight were smaller (**Figures 5A–C**). Western blot detection of xenograft tissues revealed that the protein levels of TGFBR2 and p-Smad3 were decreased in the SYTL5-silenced group compared with the control group (P<0.01) (**Figure 5D**). These results indicate that SYTL5 supports the proliferative capacity of TPC-1 cells in nude mice and is associated with activation of the TGFBR2/Smad3 signaling axis *in vivo*.

### 3.6 ZNF384 transcriptionally regulates SYTL5, inhibits its expression, and affects the malignant biological behaviors of thyroid carcinoma cells

**Figure 6:**
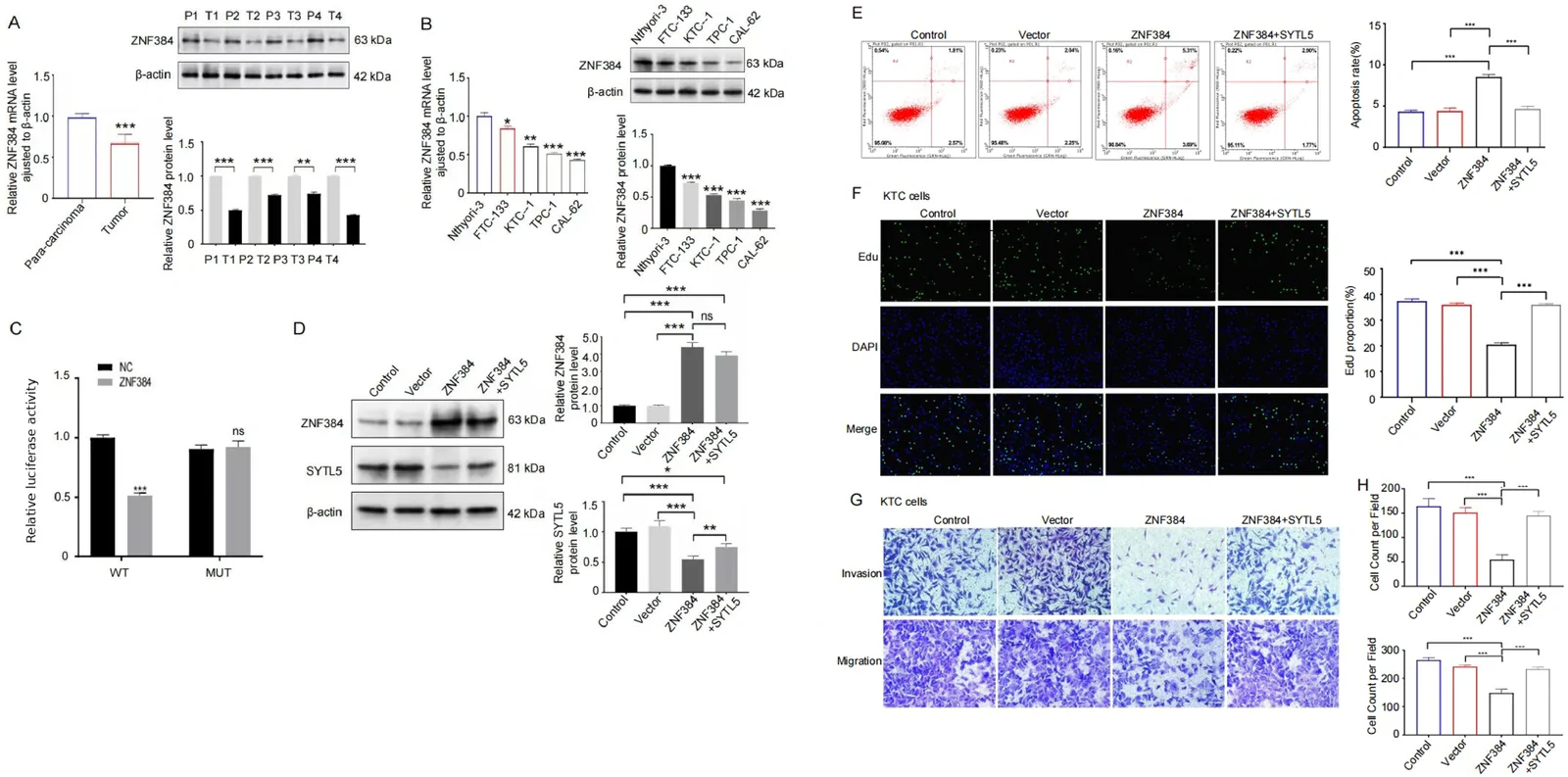
ZNF384 transcriptionally regulates SYTL5, inhibits its expression, and affects the malignant biological behaviors of KTC-1 thyroid carcinoma cells. **(A)** qPCR and Western blot detection of ZNF384 expression in fresh DTC tissues and adjacent normal tissues. **(B)** mRNA and protein expression of ZNF384 in thyroid cancer cell lines and thyroid follicular epithelial cells. **(C)** Dual-luciferase reporter gene assay verifying regulation of the SYTL5 promoter by ZNF384. **(D)** Effect of ZNF384 on SYTL5 expression in KTC-1 cells. **(E)** Effect of ZNF384 overexpression and SYTL5 rescue on apoptosis of KTC-1 cells. **(F)** Effect of ZNF384 overexpression and SYTL5 rescue on proliferation of KTC-1 cells (100×). **(G, H)** Effect of ZNF384 overexpression and SYTL5 rescue on invasion and migration of KTC-1 cells (200×). (*P<0.05, **P<0.01, ***P<0.001; ns, no statistical difference).

To clarify the expression of ZNF384 in differentiated thyroid carcinoma, qPCR and Western blot were used to detect the mRNA and protein expression of ZNF384 in fresh differentiated thyroid carcinoma tissues, adjacent normal tissues, thyroid cancer cell lines, and thyroid follicular epithelial cells. The results showed that ZNF384 expression was significantly lower in differentiated thyroid carcinoma tissues than in adjacent normal tissues (**Figure 6A**). Similarly, ZNF384 expression was significantly lower in thyroid cancer cell lines than in thyroid follicular epithelial cells (**Figure 6B**). To verify the predicted results, reporter gene plasmids containing wild-type and mutant SYTL5 promoter sequences were designed and constructed for functional verification experiments. The dual-luciferase reporter gene assay was used to verify the effect of ZNF384 on SYTL5 promoter activity. The results showed that ZNF384 overexpression significantly inhibited the luciferase activity of the wild-type SYTL5 promoter, while no significant change was observed when the predicted binding site was mutated (**Figure 6C**). This result indicates that ZNF384 can directly regulate the SYTL5 promoter region and inhibit its transcriptional activity.

To evaluate whether ZNF384 expression affects the malignant biological behaviors of differentiated thyroid carcinoma cells by regulating SYTL5, rescue experiments were performed. ZNF384 plasmids were transfected into SYTL5-overexpressed KTC-1 cells, and comparisons were made with the control group and vector group. The results showed that ZNF384 overexpression significantly reduced the protein expression of SYTL5, while concurrent overexpression of SYTL5 partially reversed the ZNF384-induced decrease in SYTL5 (**Figure 6D**). These experimental results indicate that ZNF384 has a significant regulatory effect on SYTL5 expression.

We further verified the effect of ZNF384 on the malignant biological behaviors of DTC and whether SYTL5 overexpression can attenuate these effects. Flow cytometry showed that ZNF384 overexpression significantly promoted the apoptosis of KTC-1 cells, while SYTL5 partially reversed the pro-apoptotic effect of ZNF384 (**Figure 6E**). The absolute difference was moderate, with apoptosis increasing from approximately 4.5% in control/vector cells to approximately 9% after ZNF384 overexpression, supporting a statistically significant but not dominant apoptotic contribution. EdU assays indicated that ZNF384 overexpression inhibited the proliferation of KTC-1 cells, while SYTL5 overexpression attenuated this inhibitory effect (**Figure 6F**). Transwell assays showed that the number of transmembrane KTC-1 cells was significantly decreased after ZNF384 overexpression, while the number of transmembrane cells was increased after concurrent overexpression of SYTL5 in the rescue experiment (**Figures 6G, H**). These results suggest that ZNF384 inhibits the invasion and migration of KTC-1 cells, and SYTL5 can partially attenuate this inhibitory effect.

In summary, ZNF384 downregulates SYTL5 expression by regulating the SYTL5 promoter, thereby promoting apoptosis of KTC-1 cells and inhibiting their proliferation, invasion, and migration.

### 3.7 *In vitro* experiments support the involvement of the ZNF384-SYTL5 axis in modulating TGFBR2/Smad3 signaling in DTC

**Figure 7:**
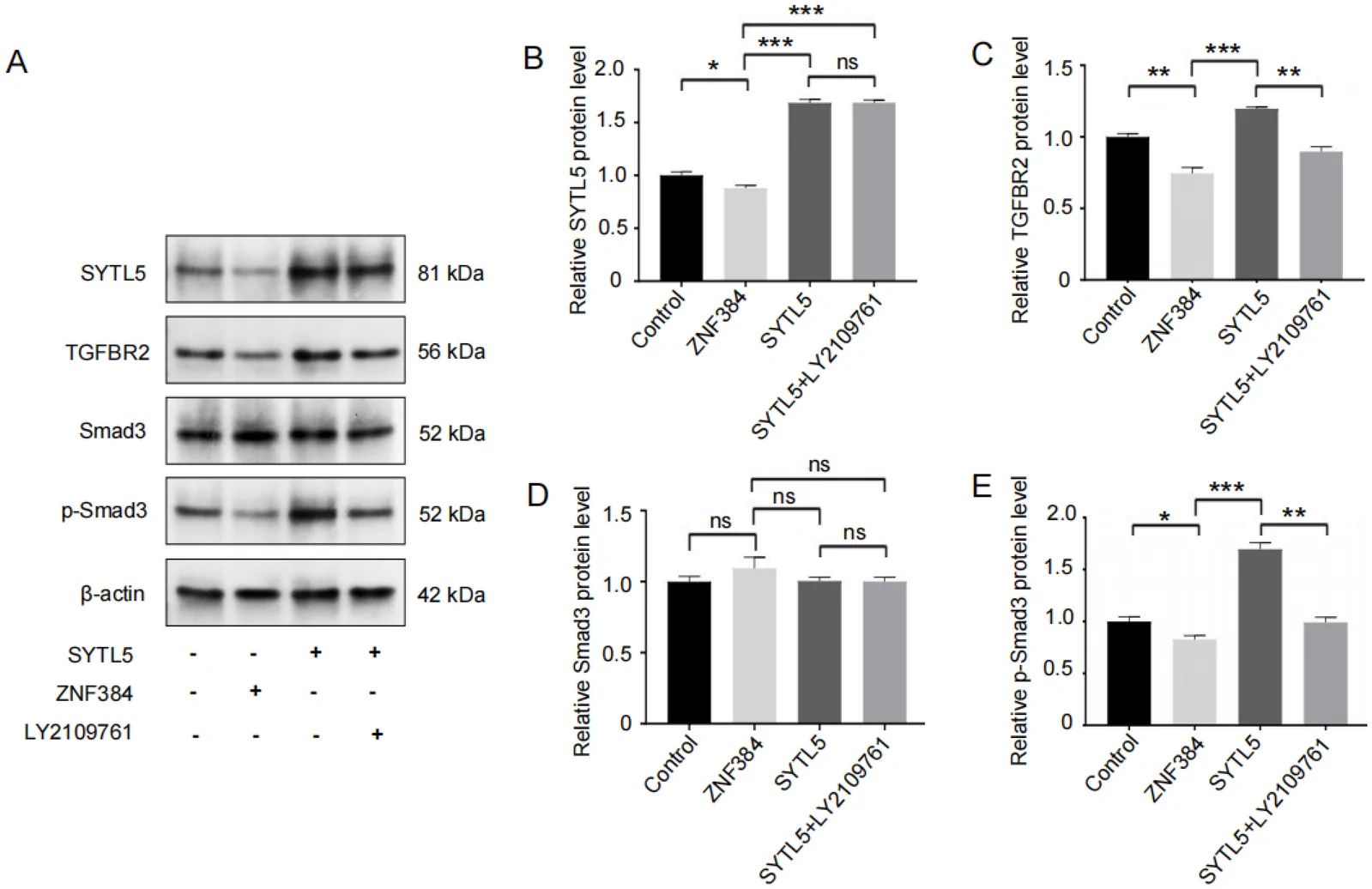
*In vitro* experiments support the mechanism by which ZNF384 targets SYTL5 to affect thyroid carcinoma via the TGFBR2/Smad3 signaling pathway. **(A)** Effect of SYTL5 overexpression combined with ZNF384 and LY2109761 on the protein expression of TGFBR2/Smad3 pathway molecules in TPC-1 cells. **(B)** Quantitative analysis of SYTL5 protein in TPC-1 cells with SYTL5 overexpression combined with ZNF384 and LY2109761. **(C)** Quantitative analysis of TGFBR2 protein in TPC-1 cells with SYTL5 overexpression combined with ZNF384 and LY2109761. **(D)** Quantitative analysis of Smad3 protein in TPC-1 cells with SYTL5 overexpression combined with ZNF384 and LY2109761. **(E)** Quantitative analysis of p-Smad3 protein in TPC-1 cells with SYTL5 overexpression combined with ZNF384 and LY2109761. (*P<0.05, **P<0.01, ***P<0.001; ns, no statistical difference).

To further explore the regulatory effect of ZNF384 on SYTL5 and downstream signaling pathways in thyroid carcinoma cells, the expression levels of key pathway proteins after ZNF384 and SYTL5 overexpression were detected. The results showed that ZNF384 overexpression downregulated the protein expression level of TGFBR2 and inhibited Smad3 phosphorylation, while SYTL5 overexpression partially reversed these effects induced by ZNF384 (**Figures 7A–E**). Treatment with the specific TGF-β pathway inhibitor LY2109761 effectively reduced the p-Smad3 activation induced by SYTL5 overexpression (**Figures 7A–E**). These results support the interpretation that ZNF384 regulates SYTL5 at the transcriptional level and that the downstream TGFBR2/Smad3 signaling state participates in the malignant biological behaviors of DTC cells. The current protein-level rescue design does not establish direct physical interaction between SYTL5 and TGFBR2, and this point is considered in the Discussion.

### 3.8 *In vivo* experiments support the involvement of the ZNF384-SYTL5 axis in DTC malignant biological behaviors via TGFBR2/Smad3 signaling

**Figure 8:**
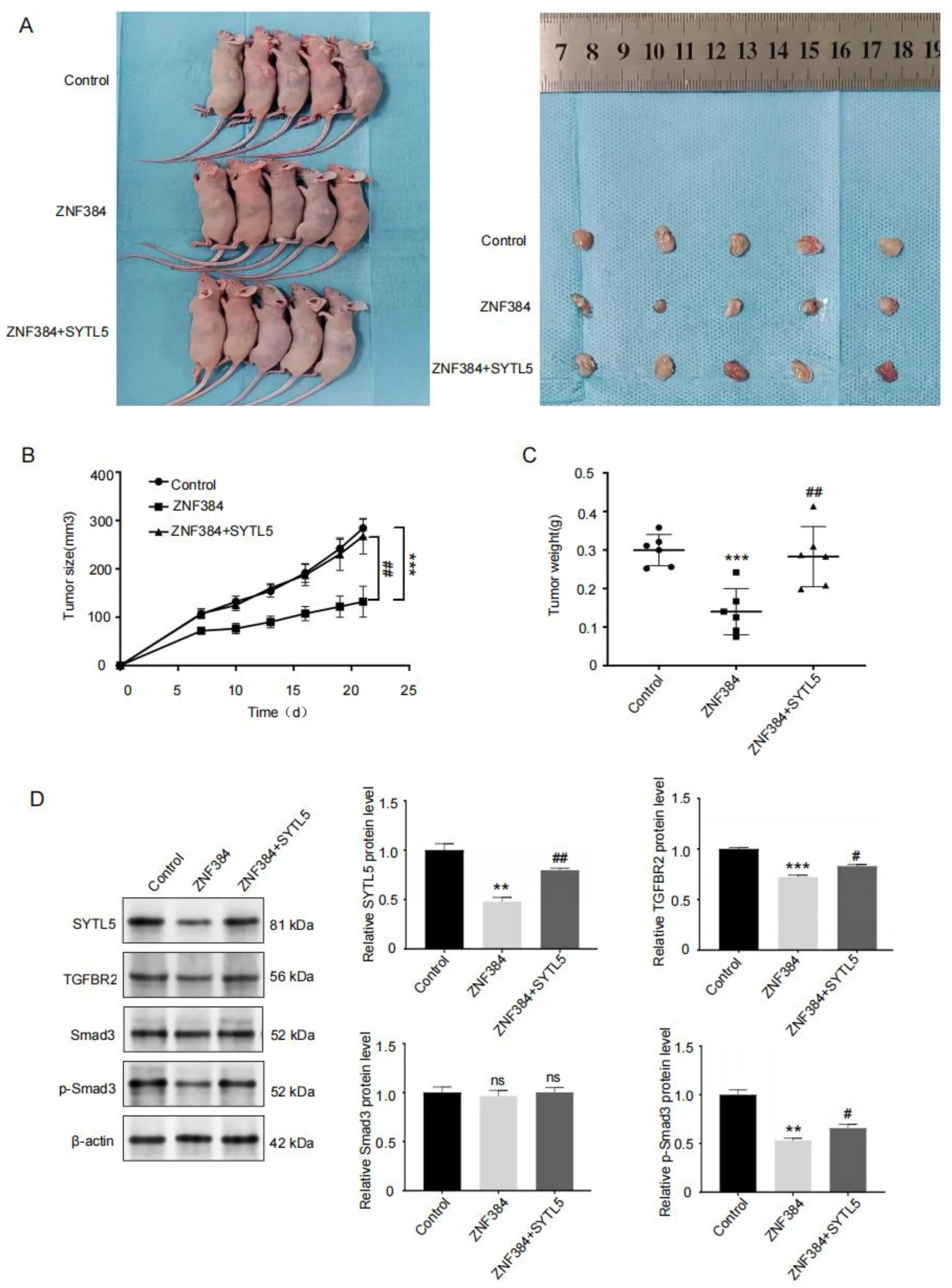
*In vivo* experiments support the involvement of ZNF384-mediated SYTL5 regulation in thyroid carcinoma proliferation and TGFBR2/Smad3 signaling. **(A)** Effect of ZNF384 overexpression on the size of nude mouse subcutaneous xenografts. **(B)** Effect of ZNF384 overexpression on the volume of nude mouse subcutaneous xenografts. **(C)** Effect of ZNF384 overexpression on the weight of nude mouse subcutaneous xenografts. **(D)** Effect of ZNF384 overexpression and SYTL5 rescue on the protein expression levels of SYTL5 and TGFBR2/Smad3 pathway molecules in nude mouse subcutaneous xenografts. (**P<0.01, ***P<0.001, compared with the control group; #P<0.05, ##P<0.01, ###P<0.001, compared with the ZNF384 group; ns, no statistical difference).

To further evaluate the effect of ZNF384 on SYTL5 and the regulation of downstream signaling pathways in DTC cells *in vivo*, three groups were established: control group, ZNF384-overexpressed group, and ZNF384-overexpressed + SYTL5-overexpressed group. TPC-1 cells, ZNF384-overexpressed TPC-1 cells, and ZNF384-overexpressed + SYTL5-overexpressed TPC-1 cells were subcutaneously injected into the axillae of nude mice, respectively. The results showed that on day 21 after inoculation, the tumor growth rate in the ZNF384-overexpressed group was significantly slower than that in the control group (P<0.01), and the average tumor volume and weight were smaller (P<0.001) (**Figures 8A–C**). In the rescue experiment group (ZNF384-overexpressed + SYTL5-overexpressed group), tumor growth was accelerated (P<0.001), and the volume and weight were increased (P<0.01) (**Figures 8A–C**). Western blot detection of xenograft tissues revealed that the protein levels of SYTL5 (P<0.001), TGFBR2 (P<0.001), and p-Smad3 (P<0.001) were decreased in the ZNF384-overexpressed group compared with the control group, and SYTL5 partially reversed the ZNF384-induced decrease in TGFBR2 and Smad3 phosphorylation (P<0.05) (**Figure 8D**).

These *in vivo* experimental results support the view that ZNF384 can suppress the TGFBR2/Smad3 signaling state and attenuate the proliferative capacity of thyroid carcinoma cells in nude mice by downregulating SYTL5 expression.

## 4 Discussion

In this study, clinical samples were collected to verify the expression of SYTL5, and cellular and animal experiments were performed to explore its role and mechanism in DTC malignant biological behaviors. SYTL5 was highly expressed in DTC tissues and cells, and high SYTL5 expression remained significantly associated with lymph node metastasis after FDR correction, whereas associations with age, sex, T stage, and TNM stage were not significant after adjustment. Distant metastasis was summarized descriptively because only one M1 patient was available. The relationship between this work and our previous SYTL5 report (6) is explicitly acknowledged: the present study extends that report by adding an independent clinical cohort, apoptosis analysis, TGFBR2/Smad3 pathway verification, ZNF384 promoter reporter assays, and *in vivo* rescue experiments. Previous studies have reported that SYTL5 promotes proliferation, invasion, and migration of the papillary thyroid carcinoma cell line K1 by upregulating RAC1 protein expression and activating the NF-κB signaling pathway (5). Using TPC-1 and KTC-1 cells, the present study further showed that SYTL5 supports proliferation, invasion, and migration and modestly suppresses apoptosis, indicating that SYTL5 acts across several malignant phenotypes rather than through a single cellular process.

From the perspective of cancer hallmarks (10), the present data most directly involve sustained proliferative capacity, resistance to cell death, and invasion/metastasis-associated behavior (11–22). The apoptosis phenotype requires cautious interpretation because the absolute changes were small and most cells remained viable; therefore, apoptosis is best interpreted as a contributory phenotype that accompanies the stronger effects of SYTL5 on proliferation and motility. The functional association between proliferation/apoptosis and invasion/migration may reflect shared upstream regulation by SYTL5-dependent signaling rather than two completely independent processes. In particular, TGF-β/Smad signaling can integrate survival, extracellular matrix remodeling, and cellular motility, providing a biologically plausible link between altered growth and enhanced invasive behavior in DTC cells.

TGF-β ligands include TGF-β1, TGF-β2, and TGF-β3, which are structurally conserved and contain a signal peptide, prodomain, and mature TGF-β domain (23). In the classical pathway, TGF-β ligands bind TGFBR2 on the cell membrane; TGFBR2 recruits and activates TGFBR1, and the activated receptor complex phosphorylates receptor-regulated Smad proteins, including Smad2 and Smad3 (24–29). Activated Smad proteins form complexes with Smad4 and translocate into the nucleus to regulate transcription. The present study focused on TGFBR2 and p-Smad3 because these proteins showed consistent changes after SYTL5 or ZNF384 manipulation, but the data do not exclude involvement of Smad2 or non-canonical pathways such as MAPK and PI3K/AKT.

TGF-β signaling has a context-dependent dual role in tumor initiation and progression (30–33). In premalignant or early malignant cells, TGF-β signaling may restrain growth, whereas in established cancers it can promote epithelial-mesenchymal transition, fibrosis, immune escape, and metastatic dissemination (34–38). In the present study, SYTL5 knockdown reduced TGFBR2 abundance and Smad3 phosphorylation, and ZNF384 overexpression produced similar changes that were partially rescued by SYTL5 overexpression. These results support the ZNF384-SYTL5-TGFBR2/Smad3 axis as a regulatory signaling module in DTC. However, because no direct binding or promoter assay was performed for TGFBR2, the manuscript does not claim that SYTL5 directly transcriptionally regulates TGFBR2. Future experiments should determine whether SYTL5 affects TGFBR2 protein stability, receptor trafficking, ligand responsiveness, or an intermediate regulator upstream of TGFBR2.

Cancer is increasingly recognized as a disease shaped by metabolic, immune, stromal, and signaling interactions (39–44). This broader context is relevant because our previous bioinformatics analysis suggested an association between SYTL5 and immune infiltration (6), while the current experiments link SYTL5 to TGFBR2/Smad3 pathway activity. Studies in thyroid carcinoma have also implicated TGF-β/Smad activity in local invasion, nodal metastasis, and pro-invasive signaling, including Smad-dependent mechanisms in aggressive thyroid cancer models (45, 46). These observations place the present findings within a thyroid cancer-specific framework and suggest that SYTL5 may contribute to a tumor state in which proliferative, invasive, and microenvironment-related signals are coupled.

Several limitations should be considered. First, overall survival and disease-free survival were not analyzed because the cohort was collected between 2023 and 2024 and mature follow-up endpoints were unavailable. Second, metastatic lymph node tissues were not included, so the expression pattern of SYTL5 in metastatic deposits could not be directly compared with matched primary tumors. Third, the *in vivo* experiments used a subcutaneous xenograft model, which enables reproducible monitoring of tumor growth and efficient tissue collection but does not fully recapitulate the thyroid-specific microenvironment; orthotopic thyroid models may better capture local invasion, thyroid stromal interactions, and metastasis-related behavior (47). Fourth, the study was gene-centered, whereas cancer progression, treatment resistance, and metastasis also involve ecological and evolutionary dynamics operating across tumor cells, stromal cells, immune cells, and tissue niches (48). Future studies incorporating orthotopic models, metastatic lymph node specimens, mature survival follow-up, Smad2/non-canonical pathway testing, and ecological-evolutionary modeling will be important for defining the translational relevance of the ZNF384-SYTL5-TGFBR2/Smad3 axis.

## 5 Conclusion

SYTL5 is highly expressed in DTC, and high expression is associated with lymph node metastasis after FDR adjustment. SYTL5 promotes DTC cell proliferation, invasion, and migration and modestly suppresses cell apoptosis. The present data support a ZNF384-SYTL5-TGFBR2/Smad3 regulatory axis in DTC, in which ZNF384 downregulates SYTL5 expression and thereby attenuates malignant biological behaviors and TGFBR2/Smad3 pathway activity. Direct regulation of TGFBR2 by SYTL5, the roles of Smad2 and non-canonical TGF-β pathways, and the clinical survival relevance of SYTL5 require additional validation. These findings suggest that SYTL5 plays a role in the tumor biological behaviors of DTC and may serve as a candidate target for future DTC treatment.

## Data Availability

The original contributions presented in the study are included in the article/**Supplementary Material**. Further inquiries can be directed to the corresponding authors.
