# Supplementary material for: SYTL5 drives malignant progression in differentiated thyroid carcinoma: unveiling its regulatory mechanisms
Source: Front Oncol. 2026 Sep 3;16:1810543. doi: 10.3389/fonc.2026.1810543 (PMC13581719; doi:10.3389/fonc.2026.1810543)
Supplement: Supplementary file 1 [file DataSheet1.pdf]

Figure 1C

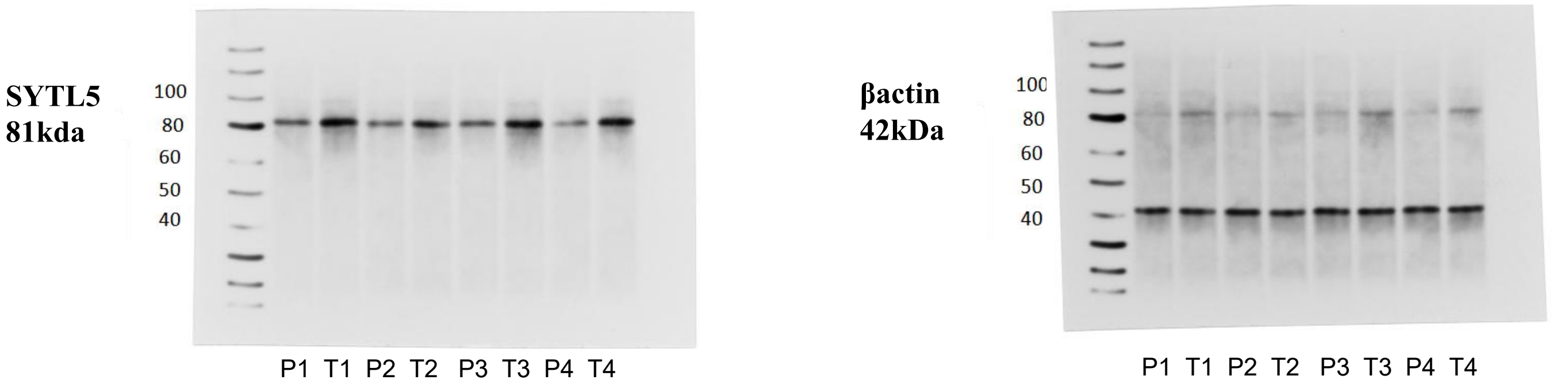

Figure 1D

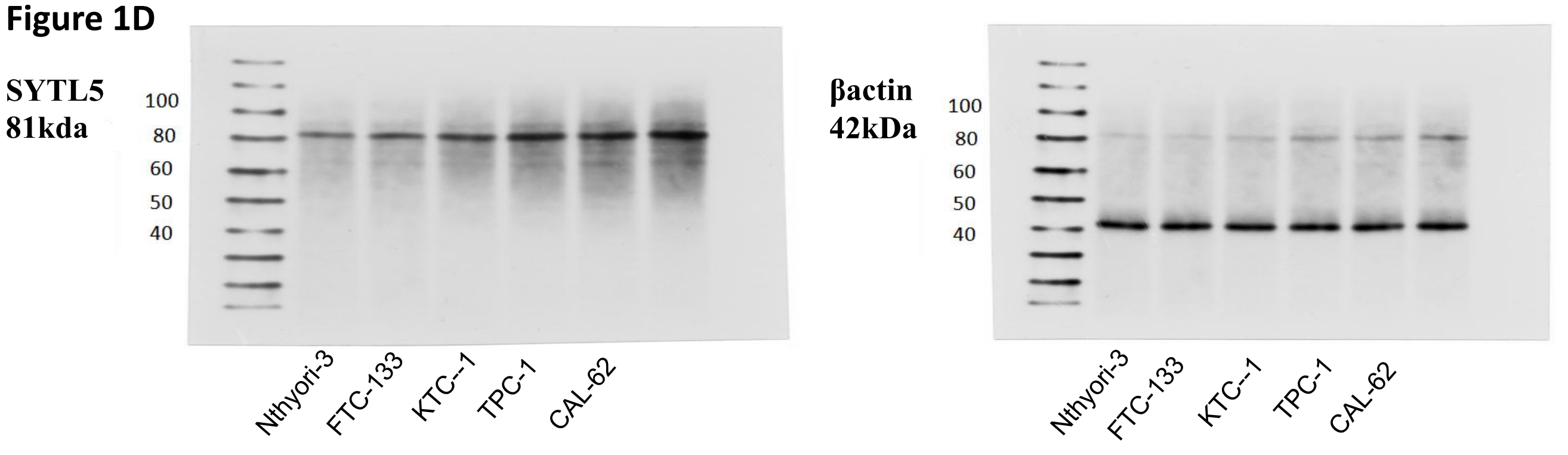

Figure 1E

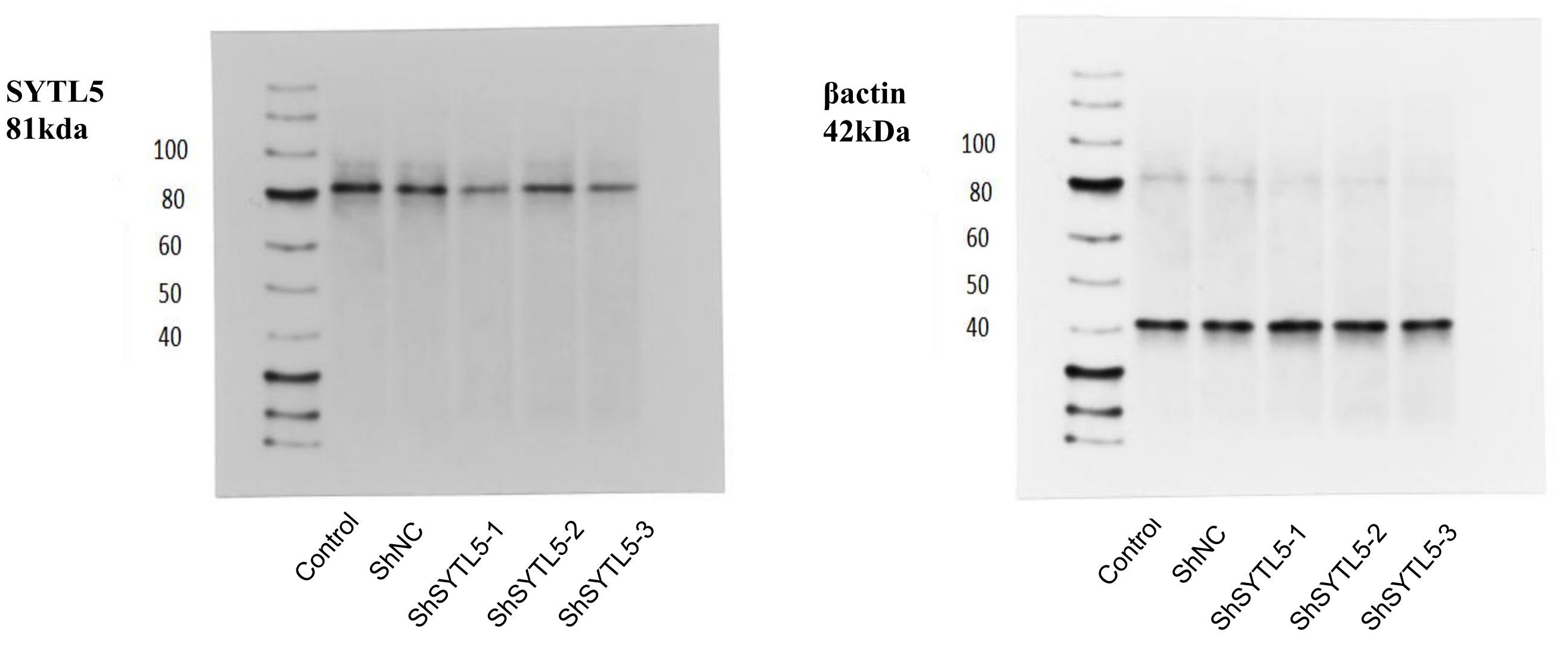

Figure 1F

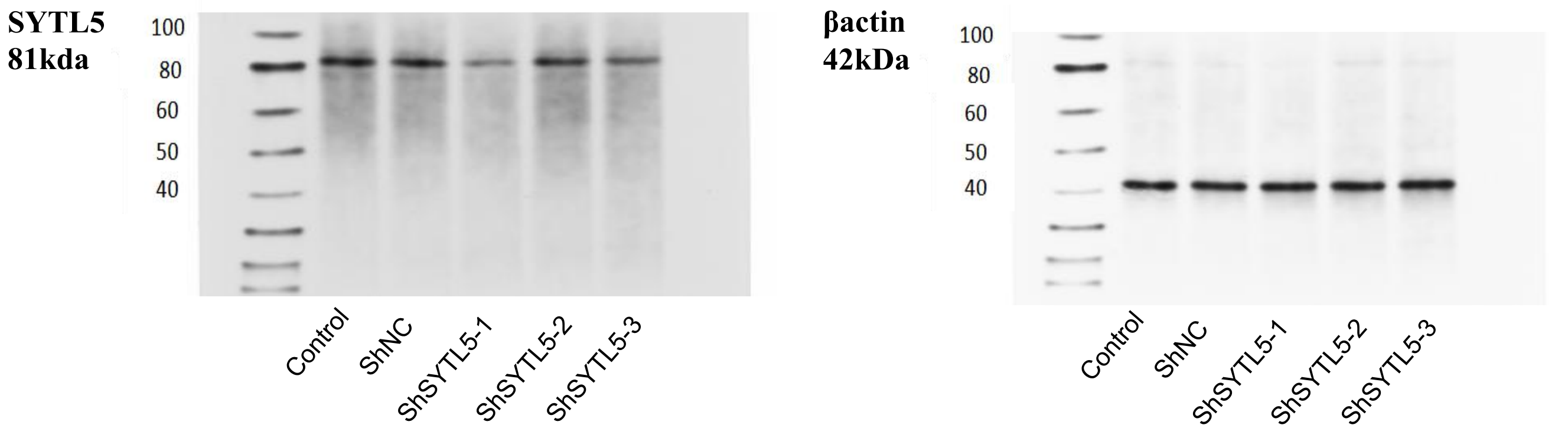

Figure 1G

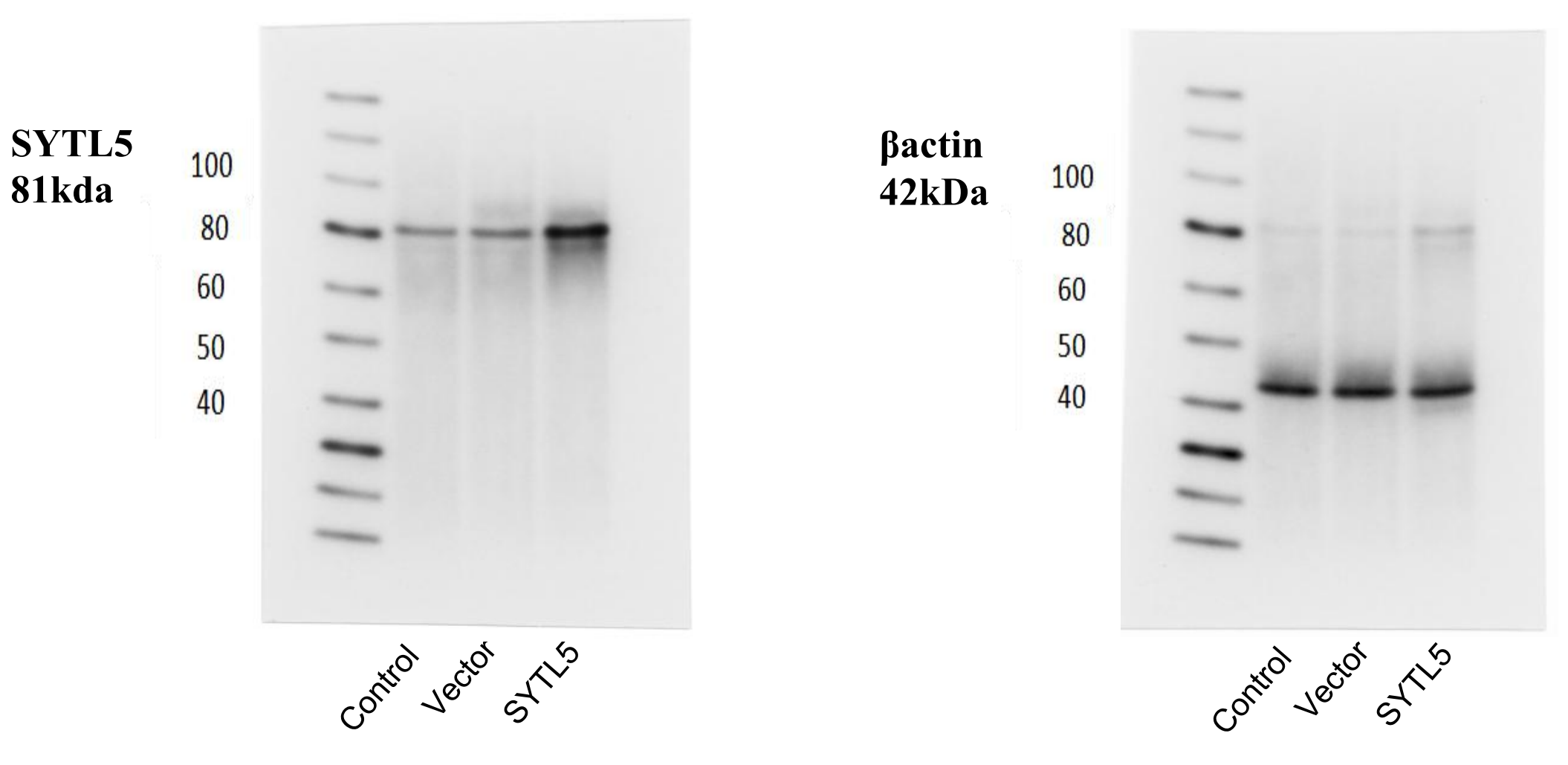

Figure 1H

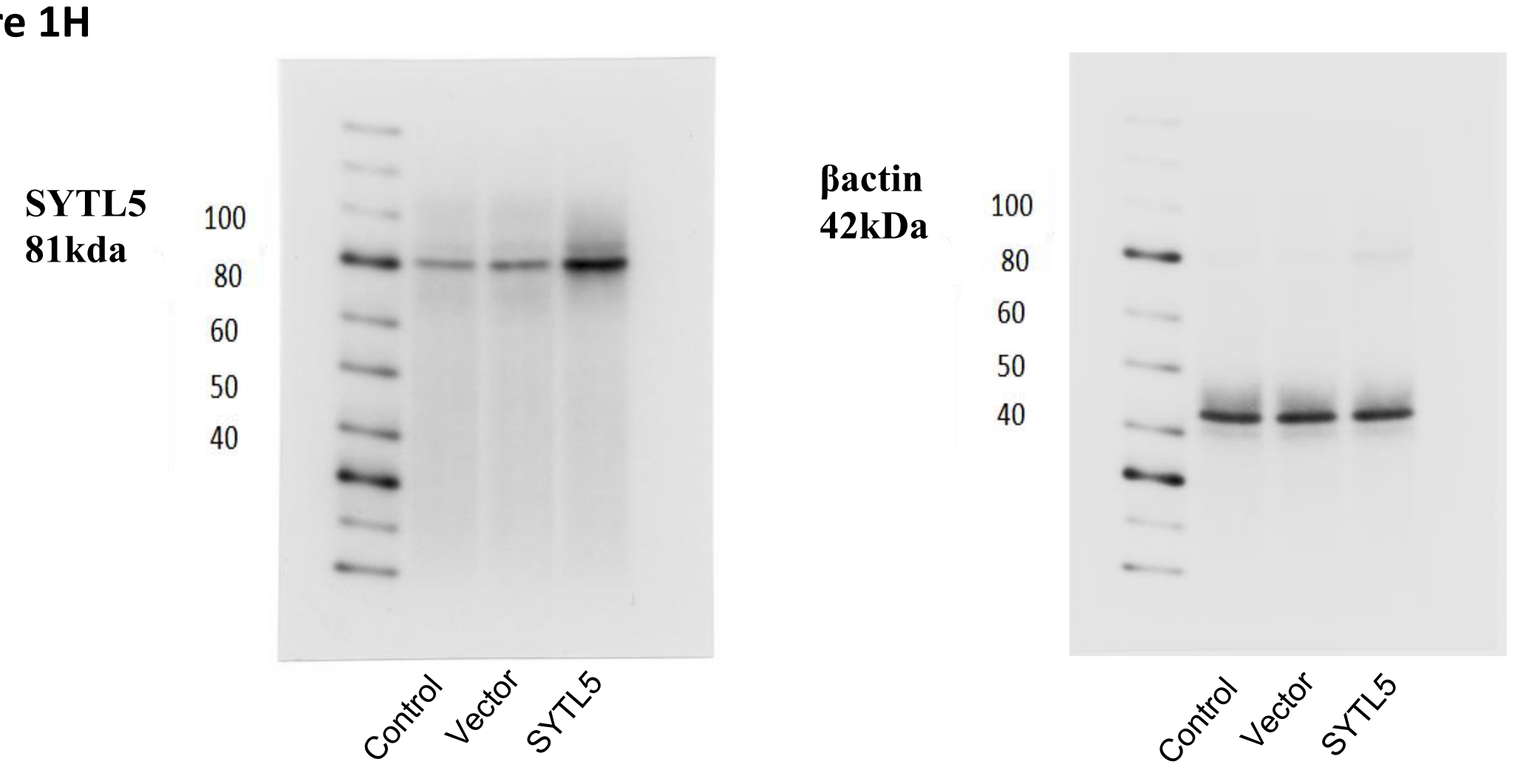

Figure 4A

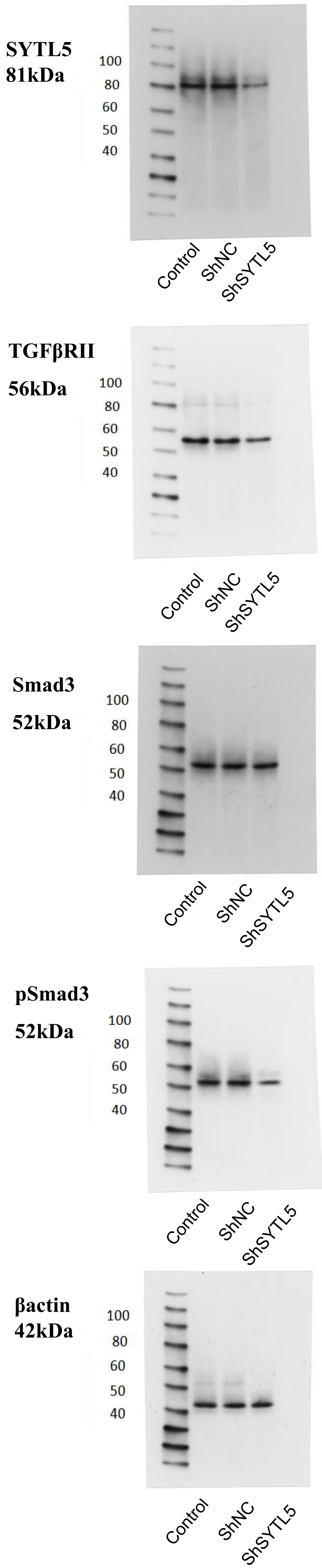
SYTL5
81kDa
100
80
60
50
40
Control
ShNC
ShSYTL5
TGFβRII
56kDa
100
80
60
50
40
Control
ShNC
ShSYTL5
Smad3
52kDa
100
80
60
50
40
Control
ShNC
ShSYTL5
pSmad3
52kDa
100
80
60
50
40
Control
ShNC
ShSYTL5
βactin
42kDa
100
80
60
50
40
Control
ShNC
ShSYTL5

Figure 5D

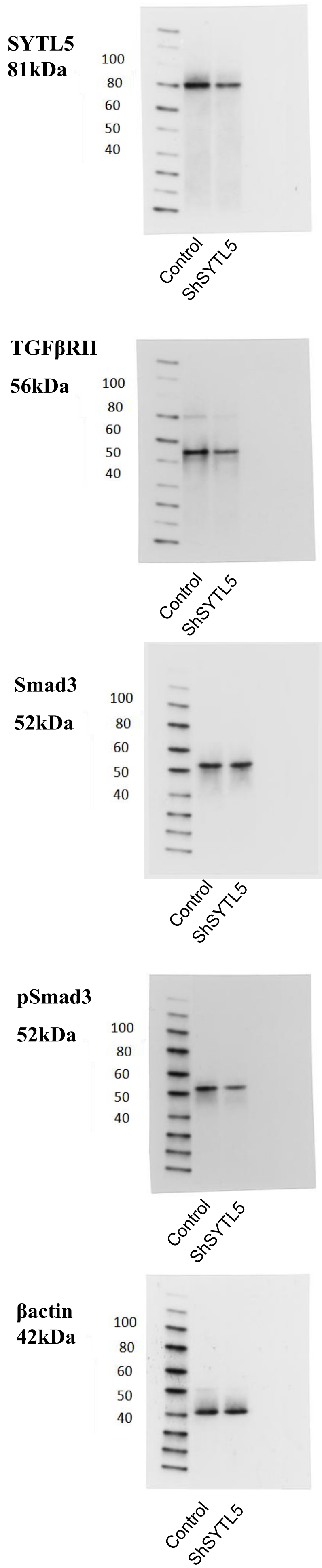

SYTL5
81kDa
100
80
60
50
40
Control
ShSYTL5
TGFβRII
56kDa
100
80
60
50
40
Control
ShSYTL5
Smad3
52kDa
100
80
60
50
40
Control
ShSYTL5
pSmad3
52kDa
100
80
60
50
40
Control
ShSYTL5
βactin
42kDa
100
80
60
50
40
Control
ShSYTL5

Figure 6A

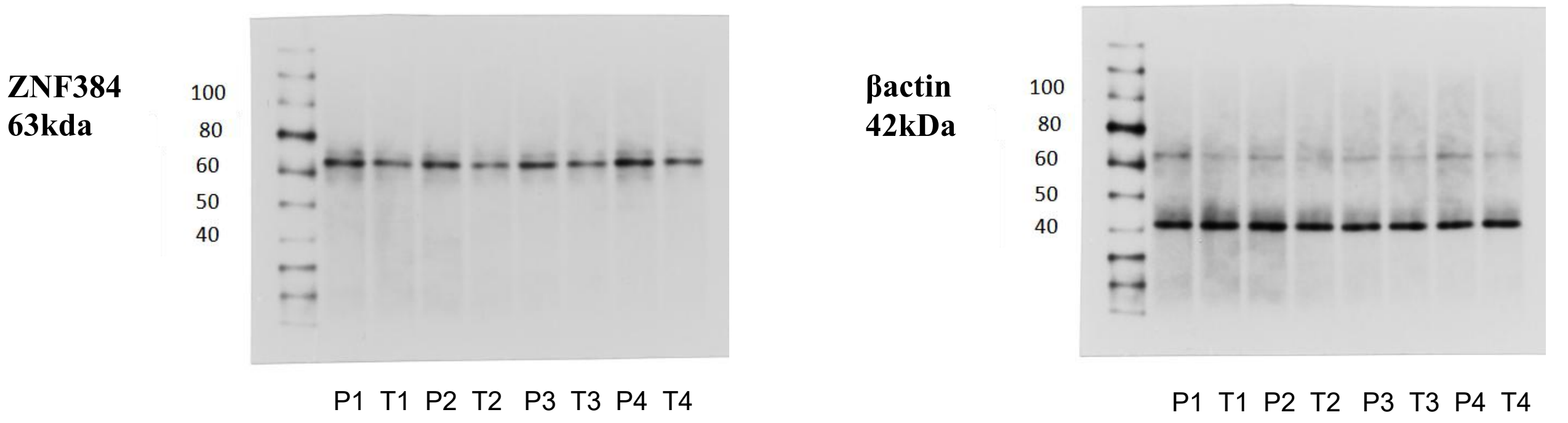

Figure 6B

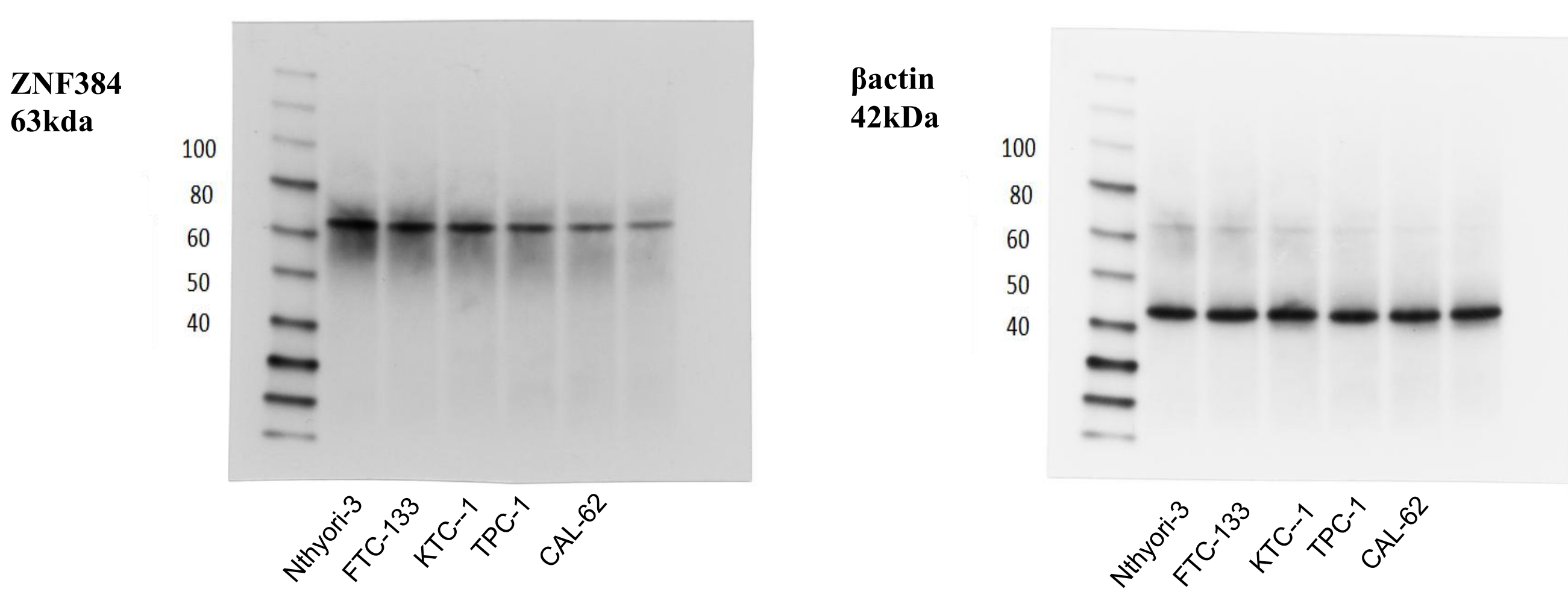

Figure 6D

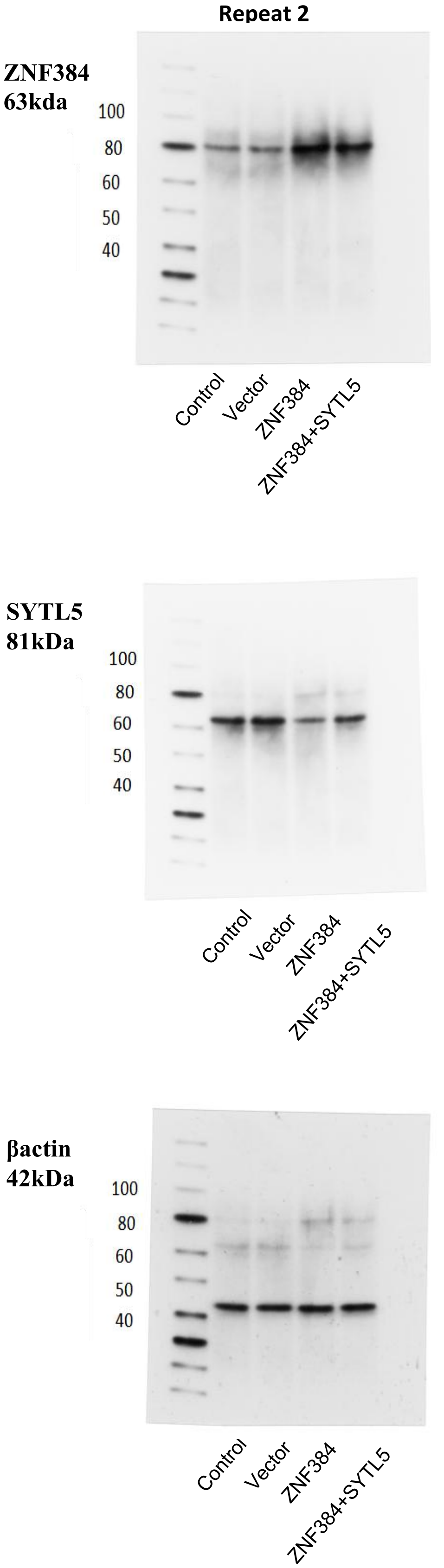
Repeat 2
ZNF384
63kda
100
80
60
50
40
Control
Vector
ZNF384
ZNF384+SYTL5
SYTL5
81kDa
100
80
60
50
40
Control
Vector
ZNF384
ZNF384+SYTL5
βactin
42kDa
100
80
60
50
40
Control
Vector
ZNF384
ZNF384+SYTL5

Figure 7A

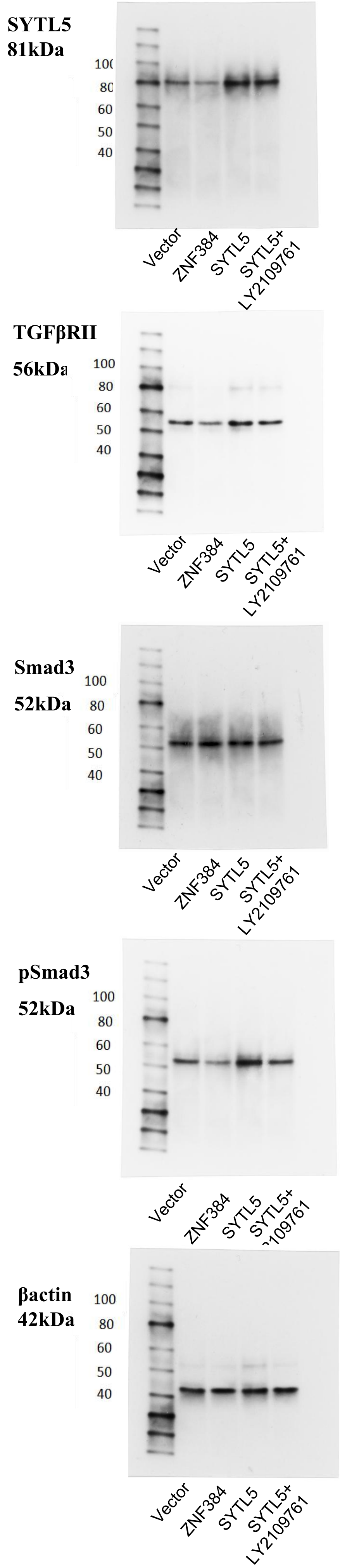
SYTL5
81kDa
100
80
60
50
40
Vector
ZNF384
SYTL5
SYTL5+
LY2109761
TGFβRII
56kDa
100
80
60
50
40
Vector
ZNF384
SYTL5
SYTL5+
LY2109761
Smad3
52kDa
100
80
60
50
40
Vector
ZNF384
SYTL5
SYTL5+
LY2109761
pSmad3
52kDa
100
80
60
50
40
Vector
ZNF384
SYTL5
SYTL5+
109761
βactin
42kDa
100
80
60
50
40
Vector
ZNF384
SYTL5
SYTL5+
LY2109761

Figure 8D

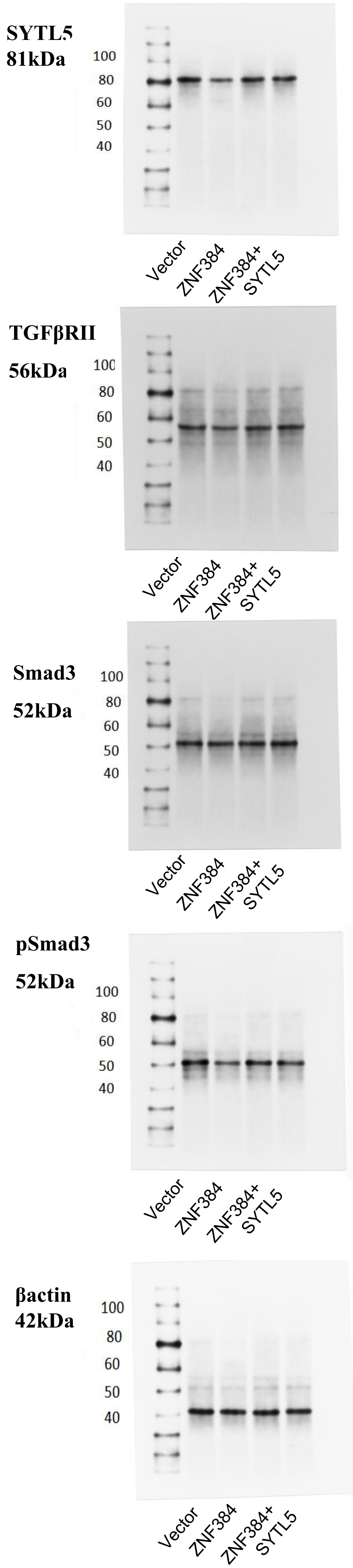
SYTL5
81kDa
100
80
60
50
40
Vector
ZNF384
ZNF384+ SYTL5
TGFβRII
56kDa
100
80
60
50
40
Vector
ZNF384
ZNF384+ SYTL5
Smad3
52kDa
100
80
60
50
40
Vector
ZNF384
ZNF384+ SYTL5
pSmad3
52kDa
100
80
60
50
40
Vector
ZNF384
ZNF384+ SYTL5
βactin
42kDa
100
80
60
50
40
Vector
ZNF384
ZNF384+ SYTL5
