# Supplementary material for: SYTL5 drives malignant progression in differentiated thyroid carcinoma: unveiling its regulatory mechanisms
Source: Front Oncol. 2026 Sep 3;16:1810543. doi: 10.3389/fonc.2026.1810543 (PMC13581719; doi:10.3389/fonc.2026.1810543)
Supplement: Supplementary file 2 [file Table1.docx]

Supplementary Table 1 Clinicopathological features of thyroid cancer with FDR-adjusted comparisons

| **Clinicopathological features** | **Number of cases** | | **SYTL5 expression** | | **P value** | **FDR-adjusted P value** |
| --- | --- | --- | --- | --- | --- | --- |
|  |  |  | **Low** | **High** |  |  |
| Age (years) | <45 | 33 | 13 | 20 | 0.7095 | 0.7171 |
|  | ≥45 | 74 | 32 | 42 |  |  |
| Gender | Male | 22 | 10 | 12 | 0.7171 | 0.7171 |
|  | Female | 85 | 35 | 50 |  |  |
| T stage | T1-2 | 92 | 36 | 56 | 0.1290 | 0.3225 |
|  | T3-4 | 15 | 9 | 6 |  |  |
| N stage | N0 | 66 | 35 | 31 | 0.0035 | 0.0175 |
|  | N1 | 41 | 10 | 31 |  |  |
| M stage | M0 | 106 | 45 | 61 | Not tested | Not tested |
|  | M1 | 1 | 0 | 1 |  |  |
| TNM stage | I-II | 104 | 43 | 61 | 0.3811 | 0.6350 |
|  | III-IV | 3 | 2 | 1 |  |  |

Note: M stage was summarized descriptively and excluded from comparative testing because the M1 subgroup contained one patient. FDR, false discovery rate.
